# Resonance Enhancement of Vibrational Polariton Chemistry Obtained from the Mixed Quantum-Classical Dynamics Simulations

**DOI:** 10.1021/acs.jpclett.3c02985

**Published:** 2023-12-06

**Authors:** Deping Hu, Wenxiang Ying, Pengfei Huo

**Affiliations:** † Center for Advanced Materials Research, Beijing Normal University, Zhuhai 519087, China; ‡ Department of Chemistry, University of Rochester, Rochester, New York 14627, United States; ¶ Institute of Optics, Hajim School of Engineering, University of Rochester, Rochester, New York 14627, United States

## Abstract

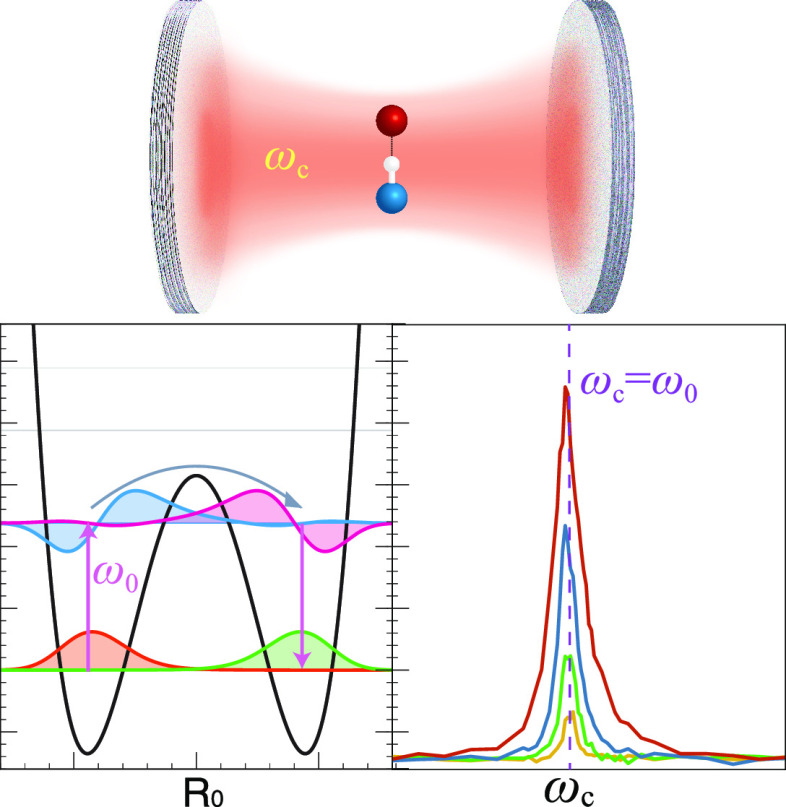

We applied a variety of mixed quantum-classical (MQC) approaches to simulate the VSC-influenced reaction rate constant. All of these MQC simulations treat the key vibrational levels associated with the reaction coordinate in the quantum subsystem (as quantum states), whereas all other degrees of freedom (DOFs) are treated inside the classical subsystem. We find that, as long as we have the quantum state descriptions for the vibrational DOFs, one can correctly describe the VSC resonance condition when the cavity frequency matches the bond vibrational frequency. This correct resonance behavior can be obtained regardless of the detailed MQC methods that one uses. The results suggest that the MQC approaches can generate semiquantitative agreement with the exact results for rate constant changes when changing the cavity frequency, the light-matter coupling strength, or the cavity lifetime. The finding of this work suggests that one can use computationally economic MQC approaches to explore the collective coupling scenario when many molecules are collectively coupled to many cavity modes in the future.

Recent experiments^1−5^ have shown that, by coupling molecular vibrations to quantized radiation modes inside an optical microcavity, the reaction rate constant can be enhanced^4,5^ or suppressed.^1−3,6^ In these experiments, there is no external influx of photons to the molecule-cavity hybrid system as the device is kept under a “dark” condition, and the change of the chemical rate constants is attributed to the formation of vibrational polaritons, quasiparticles of photon-vibrational excitation hybridization, as well as the vacuum field fluctuations.^1^ This phenomenon is referred to as the vibrational strong coupling (VSC)-enabled change of reactivities. A central feature of all VSC experiments is that when the cavity frequency (ω_c_) is resonant to the bond vibration frequency (ω_0_), i.e., when the following condition is satisfied,
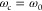
1the reaction rate constant will be enhanced or suppressed, typically up to 4–5 times compared to the rate constants outside the cavity. If we define |ν_L_⟩ as the vibrational ground state of the reactant (left well), and |ν_L_^′^⟩ as the vibrationally excited state of the reactant, then  corresponds to the frequency of the |ν_L_⟩ → |ν_L_^′^⟩ transition. This universal experimental evidence strongly suggests that the optical measurements of the vibration and the cavity modification correspond to the same quantum transition |ν_L_⟩ → |ν_L_^′^⟩, such that both the optical spectra and the VSC rate constant modification are centered around ω_0_. Indeed, the optical transition is caused by −μ̂·*E*(*t*), where μ̂ is the transition dipole operator, and *E*(*t*) is the classical laser field, whereas the molecule-cavity coupling is caused by  ∝ , where  and *â* are cavity field operators and  =  is the photonic coordinate that is proportional to the displacement field intensity inside the cavity.^7^ The shape resonance of the VSC-modified rate constant has been shown in recent quantum dynamics simulations,^8,9^ with the effect indeed maximizing according to eq 1. Unfortunately, a clear theoretical understanding of cavity-modified ground-state chemical reactivity remains missing, despite recent theoretical progress.^10^ Recent theoretical efforts have been focused on using existing rate constant theories to investigate the possible VSC modifications for the rate constant because the cavity mode is in the Infrared (IR) range of frequency (similar to regular nuclear vibrations). These explorations include the Grote–Hynes (GH) theory,^11,12^ the quantum Transition State Theory (q-TST),^13^ the Pollak–Grabert–Hänggi (PGH) theory,^14^ and numerically computing the flux-side correlation function using ring polymer molecular dynamics (RPMD).^15^ These approaches often cannot predict the correct resonant frequency that matches the quantum vibrational frequency ω_0_ measured from the optical spectra. If one describes the rate constant using classical theory, the maximum modification of the rate constant occurs when ω_c_ ≈ ω_b_, where ω_b_ is the top of the barrier frequency (imaginary frequency of the Transition State). The PGH theory,^14^ or a semiclassical version of the q-TST rate theory,^13^ finds that the cavity-frequency dependent rate modification is related to either the top of the barrier frequency ω_b_, the classical bottom of the well frequency ω_0_^cl^, or a broad frequency distribution between these two frequencies. The difficulties of using the existing rate theory to explain the VSC resonance behavior strongly suggest that the VSC mechanism does not belong to a known chemical mechanism or can be accurately described by a known analytic rate expression. Furthermore, the RPMD simulations^15^ of the VSC modification also suggest that the rate constant modification effect will maximize at ω_c_ ≈ ω_b_, raising the question of how quantum is the resonance behavior in VSC because RPMD, in principle, quantizes a DOF that includes all zero point energy and possible tunneling effects.^16^ This presents a challenge for trajectory-based quantum dynamics methods to correctly capture the resonance behavior of VSC, as well as an open question of whether the resonance VSC behavior is intrinsic quantum and thus cannot be obtained from trajectory-based simulations. We have developed an analytic theory^9^ to explain the rate constant modification and performed exact quantum dynamics simulations to assess its performance. Our theoretical investigation^9^ suggests that the VSC modification originates from the cavity mode promoting vibrational excitation |ν_L_⟩ → |ν_L_^′^⟩. Not surprisingly, it is critical to have the |ν_L_⟩ and |ν_L_^′^⟩ state description explicitly in order to have the frequency ω_0_ explicitly show up in the theory and to correctly describe the observed VSC resonance behavior. All of these hints that so long one can have the quantum state description of these key vibrational states, one should be able to obtain the correct VSC resonance condition, even for MQC approaches. The fact that the VSC-influenced dynamics is sensitive to the quantum frequency ω_0_ also explains why the GH theory,^11,12^ the PGH theory,^14^ q-TST rate theory,^13^ or the RPMD simulation^15^ cannot correctly predict the resonance condition, because these theories are often based on a partition function expression that effectively sums over all possible vibrational frequencies, and does not explicitly contain the information on ω_0_.

In this work, we used a variety of MQC approaches to simulate the VSC-influenced reaction rate constant. All of these MQC simulations treat the key vibrational levels associated with the reaction coordinate in the quantum subsystem (as quantum states), whereas all other DOFs, including photons and the cavity field as well as the associated loss bath, are the classical DOFs. We find that so long as we have the quantum state descriptions for the vibrational DOFs, one can correctly describe the VSC resonance condition in eq 1, regardless of the detailed MQC methods one used. The accuracy of the MQC approaches varies, with *k*/*k*_0_ (ratio of rate constants inside versus outside the cavity) being 3–8 times larger than the exact HEOM results. Nevertheless, all results suggest that the MQC approaches can generate semiquantitative agreement with the exact results for *k*/*k*_0_ when changing the cavity frequency (ω_c_), the light-matter coupling strength (η_c_), or the cavity lifetime (τ_c_). The finding of this work suggests that one can use computationally economic MQC approaches to explore the collective coupling scenario when many molecules are collectively coupled to many cavity modes, corresponding to the realistic experimental setup in the VSC experiments.

We use the Pauli–Fierz (PF) quantum electrodynamics (QED) Hamiltonian, which has been widely used to describe light-matter interactions in molecular cavity QED.^11^ Expressed in the dipole gauge and under the long-wavelength approximation, it is expressed as^8,9,11^
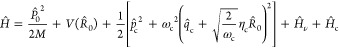
2where  is the kinetic energy of the nuclear DOF for the molecule, *M* the effective mass of the nuclear vibration,  the ground electronic state potential energy surface, and  the reaction coordinate. Furthermore,  =  and  =  are the photon mode coordinate and momentum operators, respectively, where  and *â* are the photon mode creation and annihilation operators, and ω_c_ is the cavity frequency. The light-matter coupling strength (η_c_) is expressed as η_c_ = , which characterizes the light-matter coupling strength, where ϵ_0_ is the permittivity, and  is the quantization volume inside the cavity. For simplicity, in this work, we assume that the dipole operator is linear. This has been a widely used approximation in the recent theory work.^8,11,12,17^ At the equilibrium position of the reactant coordinate *R*_0_, one can expand the dipole function using a Taylor expansion. On the other hand, the shape of these dipole functions might also significantly influence the VSC dynamics,^8,11,18^ and future work is needed to explore it. We had further explicitly assumed that the ground-state dipole moment  is linear and always aligned with the cavity polarization direction, such that .

Finally,  in eq 2 is the phonon bath, which is expressed as
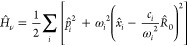
3which describes couplings between reaction coordinate  and other nuclear degrees of freedom (DOFs) , with spectral density  ≡ , and  =  is the photonic bath that describes coupling between cavity mode  and the far-field noncavity modes , giving rise to cavity loss. The cavity loss bath is also characterized by a spectral density  ≡ .

By performing harmonic analysis to the equations of motion,^19^ it is shown that the model Hamiltonian of eq 2 can be transformed (through a normal mode transformation) into the following effective Hamiltonian:^20,21^
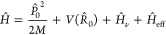
4where the cavity mode  and its associated loss bath  are combined as
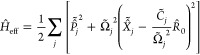
5with the effective spectral density function as  ≡ . Under the Markovian limit for the cavity loss bath , the effective spectral density can be expressed as a Brownian function,^20^
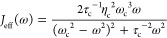
6where the broadening parameter τ_c_^-1^ is the cavity loss rate, and τ_c_ is the cavity lifetime (which is usually reported as the cavity quality factor *Q* = ω_c_τ_c_). The typical value of τ_c_ for the VSC experiments is τ_c_ ≈ 100–1000 fs.^6,22^ See Figure 1b (violet curve) for an example of *J*_eff_(ω). A detailed derivation of eq 6 can be found in ref (9).

**Figure 1:**
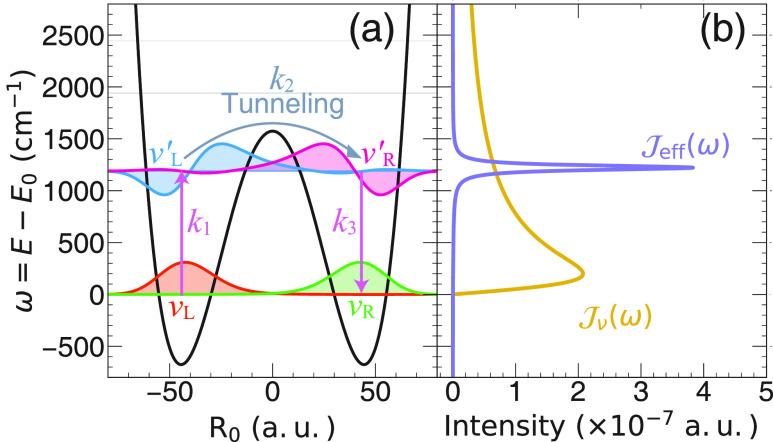
Potential energy surface for the reaction model.^8^ The pink arrows represent the thermal activation process from the vibrational ground state, |ν_L_⟩, to the vibrationally excited state, |ν_L_^′^⟩ in the reactant well (left side of the barrier). Then, through the coupling between |ν_L_^′^⟩ and |ν_R_^′^⟩, a chemical reaction occurs. Finally, the vibrational excited state |ν_R_^′^⟩ relaxed to the ground state |ν_R_⟩. The presence of the cavity mode  can be viewed as the rate-promoting vibration (RPV) mode, which explicitly enhances the transition rate of |ν_L_⟩ → |ν_L_^′^⟩. (b) The effective spectral density *J*_eff_(ω) (violet curve), corresponds to the cavity and its associated loss. The parameters are taken as the light-matter coupling strength η_c_ = 1.25 × 10^–3^ a.u., the cavity frequency *ℏω*_c_ = 1190 cm^–1^ (in resonance), and the cavity lifetime τ_c_ = 100 fs.

Figure 1 presents the first few vibrational states of the double well model, where |ν_L_⟩ denotes the vibrational ground state of the reactant (left well), |ν_L_^′^⟩ denotes the vibrationally excited state of the reactant, and similar for the product (right well). The pink arrow represents the thermal activation process from the vibrational ground state, |ν_L_⟩, to the vibrationally excited state, |ν_L_^′^⟩ in the reactant well. Then, through the coupling between |ν_L_^′^⟩ and |ν_R_^′^⟩, a chemical reaction occurs. Finally, the vibrational excited state |ν_R_^′^⟩ relaxes to the ground state of the product |ν_R_⟩. The presence of the cavity mode  can be viewed as the rate-promoting vibration (RPV) mode, which explicitly enhances the transition rate of |ν_L_⟩ → |ν_L_^′^⟩. The symmetric double-well model^8^ is used to model the reaction, with details given in Section I in the Supporting Information. We have used the exact quantum dynamics simulation (HEOM approach) to check that the *k*/*k*_0_ semiquantitatively converges in the four states subspace (see Section II in the Supporting Information).

In our previous work using the exact HEOM simulations,^9^ we have identified the reaction mechanism outside the cavity as follows:
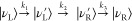
7

Note that this is the quantum description of the reaction based on quantized states, whereas the classical description is a barrier crossing along the reaction coordinate. The mechanism for the reaction is that the thermal activation process causes the transition of |ν_L_⟩ → |ν_L_^′^⟩. Then, the reaction occurs through the diabatic couplings between |ν_L_^′^⟩ and |ν_R_^′^⟩, followed by a vibrational relaxation of the product state, |ν_R_^′^⟩ → |ν_R_⟩. The rate-limiting step for the entire process is *k*_1_, where *k*_2_ ≫ *k*_1_, such that the populations of both |ν_L_^′^⟩ and |ν_R_^′^⟩ reach a steady state (plateau in time), and from the steady-state approximation, the overall rate constant for the reaction is *k*_0_ ≈ *k*_1_ (see Appendix B in ref (9) for detailed discussion).

Upon coupling to the cavity, we found that the role of the cavity field is to promote the |ν_L_⟩ → |ν_L_^′^⟩ transition, causing a larger steady-state population of the |ν_L_^′^⟩ and |ν_R_^′^⟩ states. As such, the cavity mode  acts like a rate-promoting vibrational mode,^23^ causing the rate constant enhancement. We further assumed that the rate constant inside the cavity *k* can be decomposed as *k* ≈ *k*_1_ = *k*_0_ + *k*_VSC_, where *k*_0_ is the rate constant outside the cavity, and *k*_VSC_ is the rate constant enhancement caused by coupling to the cavity. Note that the effect of the cavity causes an enhancement of *k*_1_, which is the vibrational excitation process, and because this step is rate-limiting, the effect of the cavity thus manifests in the entire apparent rate constant. With this observation, we have derived an analytic expression for the cavity-enhanced part of the rate constant for the transition |ν_L_⟩ → |ν_L_^′^⟩ (with a frequency ω) expressed as follows:^9^
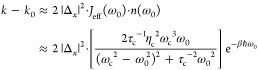
8where  is the transition dipole matrix element associated with the |ν_L_⟩ → |ν_L_^′^⟩ transition. Equation 8 has a clear resonance behavior at ω_c_ = ω_0_ due to the effective spectral density *J*_eff_(ω_0_) (cf. eq 6). In eq 8, we have assumed that the phonon coupling in  (eq 3) contributes to *k*_0_, and the cavity photon bath coupling in  (eq 5) contributes to *k*_VSC_, such that they are additive and give *k* = *k*_0_ + *k*_VSC_. This is indeed the case under the golden rule limit for both *k*_0_ and *k*_VSC_, and should be viewed as a fundamental approximation beyond the golden rule limit. The analytic rate expression produces semiquantitative agreement with the exact HEOM simulations,^9^ but it needs to be further corrected with phonon broadening (see Appendix A in ref (9)) when τ_c_ → *∞*.

These early mechanistic studies suggest that the key to obtaining the correct resonance behavior in *k*/*k*_0_ is to have the *quantum description* of the vibrational state |ν_L_⟩ and |ν_L_^′^⟩, such that the quantum vibrational frequency ω_0_ ≡  is explicitly incorporated in the simulations. The quantum vibrational frequency ω_0_ in the current model is different than the barrier frequency ω_b_ or the classical vibrational frequency. Note that, in most of the TST-type rate theory or RPMD simulations, there is no explicit information on ω_0_, and the partition function, in principle, contains all vibrational frequencies. This is the reason why these existing rate theories cannot give rise to the correct resonance condition in eq 1. The recently derived analytic theory in eq 8, on the other hand, explicitly contains ω_0_ and, thus, is capable of correctly describing the correct resonance condition.

In this work, we use a variety of MQC dynamics methods to investigate the resonance behavior of VSC. Note that various MQC approaches (Ehrenfest dynamics and SQC) have been applied to simulate mixed quantum-classical electrodynamics,^24^ which helps to effectively describe spontaneous emission processes.^25^ In particular, we use eq 4 as the Hamiltonian and include only the key vibrational states in eq 7 as our quantum subsystem and all of the other DOFs as the classical DOFs. This means that, in the vibration subspace |*i*⟩∈{|ν_L_⟩, |ν_L_^′^⟩, |ν_R_^′^⟩, |ν_R_⟩} (which are the diabatic states), we use projection operator  to represent the model potential  in eq 4 as follows:
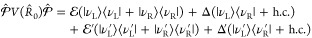
9where  and  are vibrational energy for diabatic states {|ν_L_⟩, |ν_R_⟩} and {|ν_L_^′^⟩, |ν_R_^′^⟩}, respectively, and Δ and Δ′ are the diabatic couplings between |ν_L_⟩ and |ν_R_⟩, as well as between |ν_L_^′^⟩ and |ν_R_^′^⟩, respectively. The quantum vibrational frequency is , which corresponds to the frequency of the |ν_L_⟩ → |ν_L_^′^⟩ transition. This subspace is also used to evaluate the coupling operators between the quantum DOF  and the classical DOF , including  (from  in eq 3) and  (from  in eq 5), with the detailed expressions in Section I in the Supporting Information. These quantum-classical couplings include both diagonal terms (Holstein coupling) and off-diagonal terms (Peierls couplings). Furthermore, in a truncated matter subspace, one must ensure that all operators are properly confined in the same truncated electronic subspace,^26^ in order to generate consistent and meaningful results. For the PF Hamiltonian (eq 2) under the dipole gauge, one must truncate the dipole self-energy term  consistently in the subspace . This should be done as  (which is what we used in this work) and not as . Denoting the electronic Hilbert space identity as , where  contains the adiabatic electronic states outside the subspace defined by , then  is properly confined in the subspace , whereas  contains the terms outside the subspace .

The nuclear vibrational DOF in  is treated classically, with a model spectral density
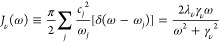
10where γ_ν_ = 200 cm^–1^ and λ_ν_ = 0.1ω_b_γ_ν_/2, according to the previous work in ref (8). The frequency of bath modes ω_*j*_ and the coupling strength *c*_*j*_ are sampled based on a numerical procedure that discretizes the *J*_ν_(ω) discussed in ref (27) (or in general, the algorithm described in ref (28)), with 300 bath modes and the maximum bath frequency ω_m_ = 2000 cm^–1^. Furthermore, we treat cavity mode  and its loss bath  together, described by eq 5, characterized by *J*_eff_(ω) in eq 6. As such, the cavity mode and the lossy environment are described by the normal modes . We sample the frequency  and the coupling strength , according to *J*_eff_(ω) in eq 6 with an equally spaced sampling strategy.^29^ The bath frequencies  are near the peak value ω_c_. The initial conditions of the classical DOFs {*p*_*i*_, *x*_*i*_} (phonon vibrational modes in , eq 3) and  (photonic normal modes in , eq 5) are sampled based on their Wigner distributions. Computational details are provided in the Section III in the Supporting Information.

We use the symmetrical quasi-classical (SQC) window function approach (γ-SQC),^30^ the linearized semiclassical spin mapping approach (spin-LSC),^31,32^ Ehrenfest dynamics,^33^ and the global-flux surface hopping (GFSH) approaches^33−36^ as the MQC method to simulate the vibrational quantum dynamics influenced by the cavity. A brief outline of each method is also provided in Section III in the Supporting Information. The reaction rate constant is obtained by fitting the population dynamics of |ν_R_⟩ state,^37,38^ with the details provided in Section IV in the Supporting Information. The initial conditions for the quantum DOFs are method-specific, as described in Section III in the Supporting Information, and the initial conditions for the classical DOF (including the phonon bath and the effective photonic bath) ) are sampled from the corresponding thermal Wigner distributions. Further details of the MQC simulations are provided in Section V in the Supporting Information. The rate constant from the HEOM simulations is obtained using the procedure detailed in ref (8) or ref (9), with details provided in Section II in the Supporting Information.

Figure 2 presents the population dynamics of the vibrational states obtained from the γ-SQC approach,^30^ for outside the cavity case (thick solid lines) and for coupling inside a resonant cavity (thin lines with open circles), with a coupling strength η_c_ = 1.25 × 10^–3^ a.u. The cavity lifetime is τ_c_ = 2000 fs. In Figure 2a, one can clearly see that when coupling to the cavity, the population of the |ν_L_⟩ state (red) decays much faster, compared to the outside cavity scenario, and the population of |ν_R_⟩ arises accordingly faster when coupling the molecule inside the cavity. Examining the population dynamics of the excited states of |ν_L_^′^⟩ (blue) and |ν_R_^′^⟩ (magenta) also reviews that, upon coupling to the cavity, the steady-state populations of these vibrationally excited states are enhanced compared to the outside cavity case, in agreement with our recent results^9^ that leads to a successful analytic rate constant in eq 8. Population dynamics obtained from the exact HEOM approach are also provided in Section II in the Supporting Information. Population dynamics obtained from other MQC methods, including spin-LSC, Ehrenfest dynamics, and GFSH are provided in Section III in the Supporting Information. Despite qualitatively capturing the same mechanism, all of the trajectory-based methods seem to overestimate the absolute value of the rate by a factor of 10 or more, even outside the cavity case. This is likely due to the less accurate trajectory treatment of the classical DOF,^39^ especially for the off-diagonal Peierls couplings terms  (from  in eq 3) (see additional discussions in Section VI in the Supporting Information. Future investigations will focus on further improvement of the vibrational dynamics using MQC methods.

**Figure 2:**
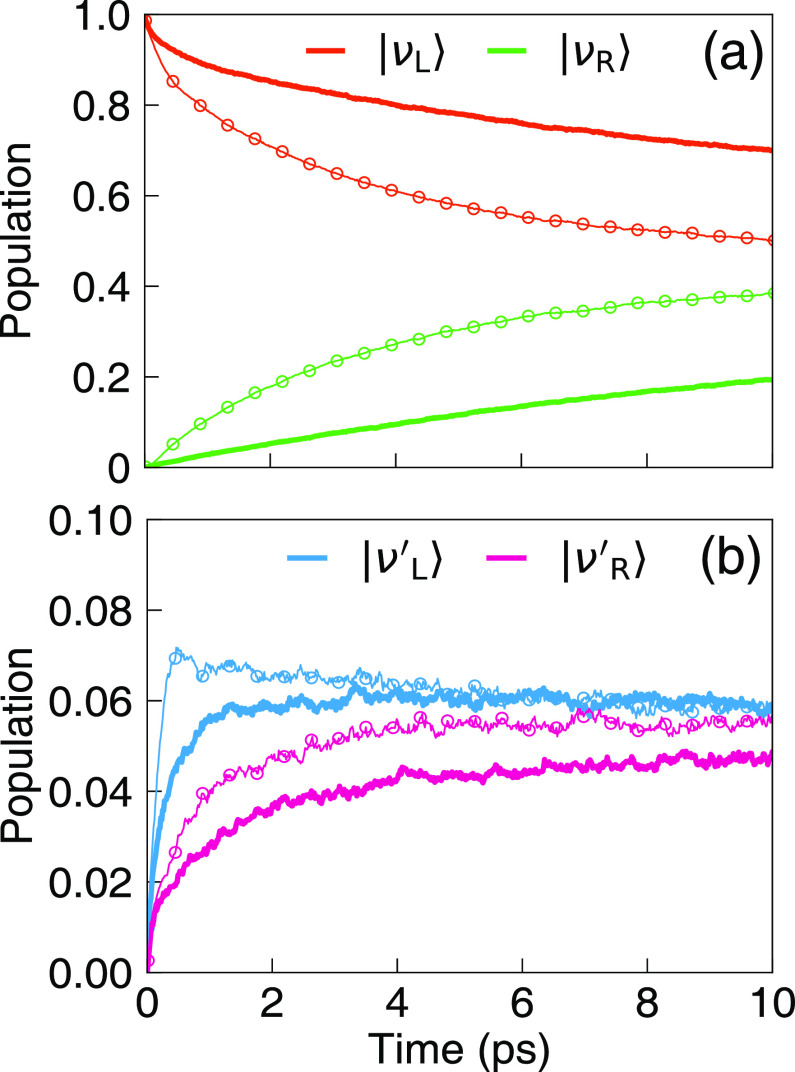
Population dynamics of the four key vibrational states, with (a) |ν_L_⟩ (red) and |ν_L_⟩ (green), and (b) |ν_L_^′^⟩ (blue) and |ν_R_^′^⟩ (magenta). Thick solid lines represent the dynamics that occur outside the cavity, and thin lines with open circles represent the dynamics that occur inside the cavity with a coupling strength η_c_ = 1.25 × 10^–3^ a.u. The cavity lifetime is τ_c_ = 2000 fs.

Figure 3 presents the profile of the resonant VSC rate constant enhancement *k*/*k*_0_ as a function of cavity frequency ω_c_ with different light-matter coupling strengths η_c_ (color-coded with the legend in Figure 3a). Figure 3a presents the results obtained from the exact HEOM simulations, and Figure 3b presents the results obtained from γ-SQC. The most important feature of the γ-SQC is that it successfully reproduces the correct resonance condition (eq 1), maximizing the rate constant at the frequency ω_c_ ≈ ω_0_, and exhibiting a sharp peak that is semiquantitatively similar to the HEOM results in Figure 3a. More importantly, it is not relevant to barrier frequency ω_b_. Thus, the current simulation using γ-SQC provides a significant improvement in terms of predicting the correct resonance VSC behavior, compared to the previous RPMD simulations, which predict an incorrect resonance condition at ω_c_ ≈ ω_b_ (see Figure 2 of ref (15)). On the other hand, the magnitude of the *k*/*k*_0_ from the γ-SQC simulations seems to be three to four times larger than the corresponding values of HEOM. Despite being less ideal in terms of predicting the precise value of the *k*/*k*_0_, it is encouraging to see that by treating |ν_L_⟩ and |ν_L_^′^⟩ as quantum states in the MQC simulation (and even treat cavity mode as part of the classical DOFs), one can still recover the correct VSC resonance condition.

**Figure 3:**
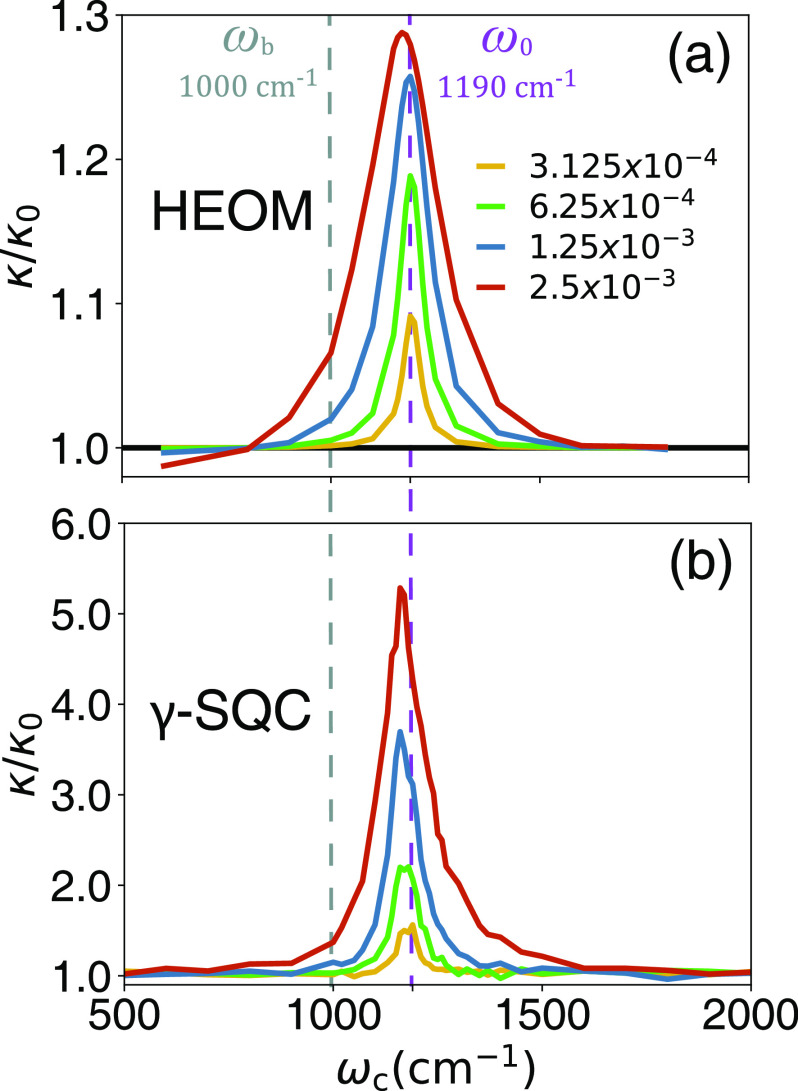
Resonance behavior of *k*/*k*_0_ as a function of cavity frequency ω_c_ with different light-matter coupling strengths η_c_ (color-coded with the legend in panel (a)), obtained from (a) the numerically exact HEOM simulation and (b) γ-SQC simulations. The cavity lifetime is set to be τ_c_ = 2000 fs.

Indeed, γ-SQC is one of the best trajectory-based approaches and has been shown to outperform Ehrenfest dynamics and trajectory surface hopping approaches in model systems,^30^ in ab initio dynamics simulations,^40^ and in exciton-polariton dynamics for a model system.^41^ The natural question is then, was the success in Figure 3 only belongs to γ-SQC as a superior MQC method or it is more general for any MQC method, as long as we include the key vibrational states inside the quantum subsystem? To answer this question, we perform additional MQC simulations to obtain *k*/*k*_0_ using various other approaches.

Figure 4 presents *k*/*k*_0_ obtained from various other MQC methods and compares them with the HEOM results in Figure 4a. These MQC methods include the Ehrenfest dynamics (Figure 4b), GFSH (Figure 4c), and the spin-LSC approach (Figure 4d). As one can clearly see, all of these trajectory-based methods semiquantitatively capture the correct VSC resonance behavior, where the maximum of the rate constant enhancement occurs at ω_c_ ≈ ω_0_. Of course, some methods are more accurate than others in terms of the quantitative accuracy of *k*/*k*_0_, where GFSH seems to be the least accurate and spin-LSC seems to be providing more accurate values of *k*/*k*_0_ that is consistent with γ-SQC (see Figure 3), although exhibiting some slightly negative populations (see Figure S6 in the Supporting Information) due to the feature of this mapping-based approach. These results confirm our hypotheses that the correct VSC resonance condition (eq 1) will emerge as long as one includes the key vibrational states inside the quantum subsystem.

**Figure 4:**
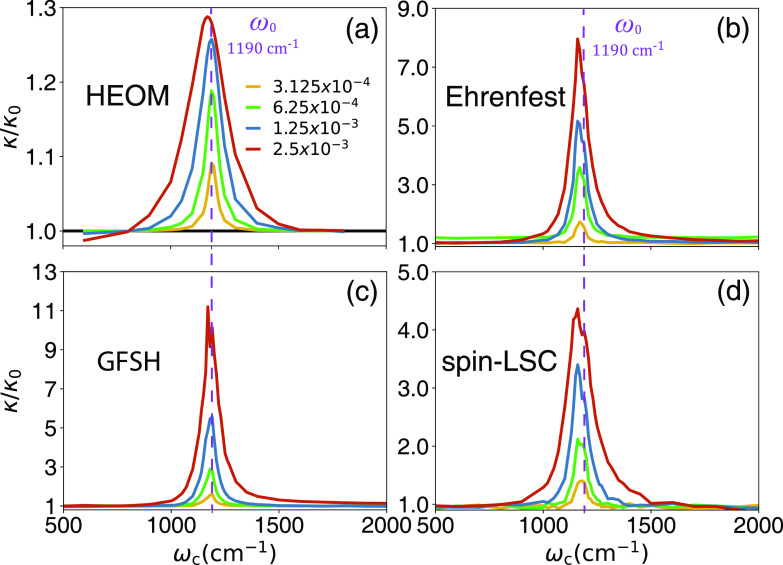
Resonance behavior of *k*/*k*_0_, as a function of cavity frequency ω_c_, with different light-matter coupling strengths η_c_ (color-coded with the legend in panel (a)). The results are obtained from (a) the numerically exact HEOM simulation, (b) Ehrenfest dynamics, (c) the Global-Flux Surface Hopping (GFSH) approach, and (d) the spin-LSC method. The parameters are exactly the same as those in Figure 3.

Figure 5 presents *k*/*k*_0_ as a function of cavity frequency ω_c_ with different cavity lifetime τ_c_ (color-coded with the legend in Figure 5a), obtained from the numerically exact HEOM simulation (Figure 5a) and γ-SQC simulations (Figure 5b). The light–matter coupling strength is η_c_ = 1.25 × 10^–3^ a.u., and the cavity lifetime is varied from 100 to 10000 fs. We can see the VSC enhancement of the *k*/*k*_0_ increase first, from τ_c_ = 100 fs (black dashed lines) to τ_c_ = 200 fs (red solid line), then gradually decay by further increasing the cavity lifetime to τ_c_ = 500 fs and more. This rate constant turnover was discovered in our previous work,^9^ and here, we demonstrate that γ-SQC (and, in fact, all MQC methods explored here) can semiquantitatively reproduce the same trend. The analytic theory in eq 8 cannot describe such a turnover of *k*/*k*_0_, as a function of τ_c_, and future analytic work is required to develop a theory that is capable of describing this behavior. We should note that the analytic theory in eq 8 cannot explain such turnover, as investigated in ref (9). This turnover, in terms of the τ_c_^–1^, can be viewed as a Kramers type of turnover, where τ_c_^–1^ is the friction parameter between the cavity mode  and the photon loss bath. Future theoretical work is needed to build an analytic theory to explain such an effect, but all of the MQC approaches that we have explored here can already successfully explain such a turnover.

**Figure 5:**
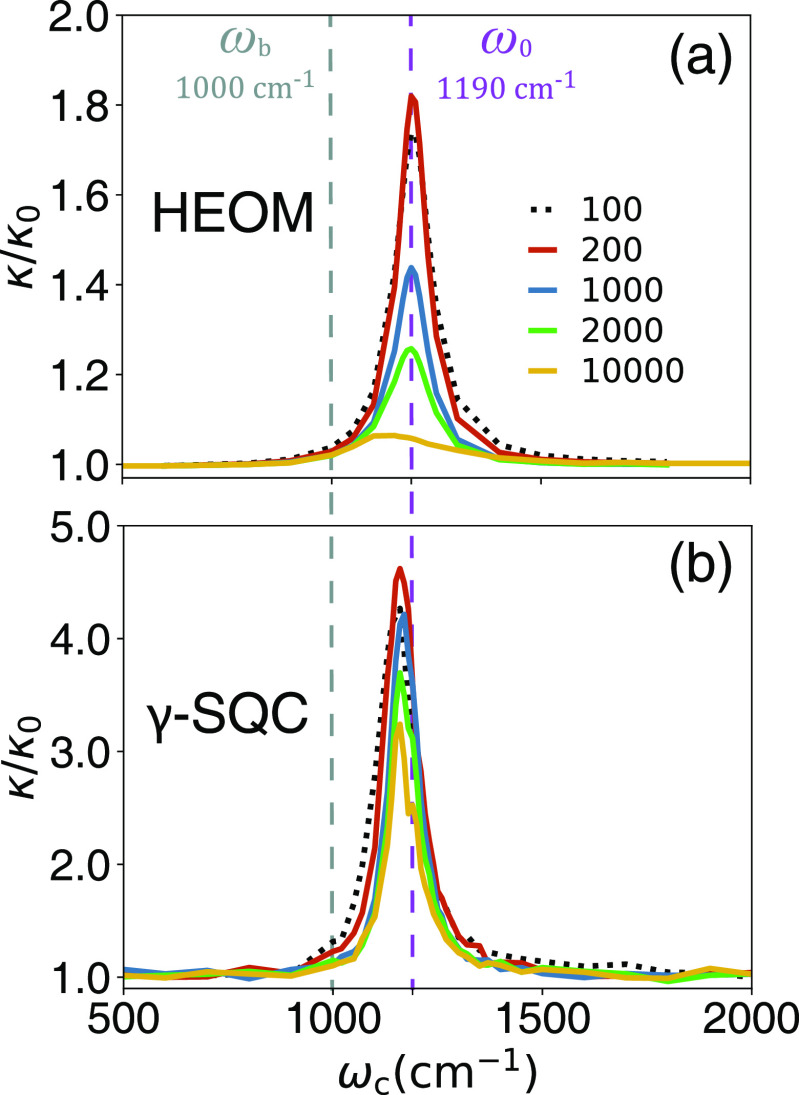
Resonant VSC rate constant *k*/*k*_0_ as a function of cavity frequency ω_c_ with different cavity lifetime τ_c_ (color-coded with the legend in panel (a)), obtained from (a) the numerically exact HEOM simulation and (b) γ-SQC simulations. The light–matter coupling strength is η_c_ = 1.25 × 10^–3^ a.u.

To summarize, we applied a variety of MQC approaches to simulate the VSC-influenced reaction rate constant. These MQC simulations treat the key vibrational levels associated with the reaction coordinate in the quantum subsystem (as quantum states), whereas all other DOFs are treated inside the classical subsystem. We find that as long as we have the quantum state descriptions for the vibrational DOFs, one can correctly describe the VSC resonance condition in eq 1, regardless of the specific MQC methods that one used. The results suggest that the MQC approaches can generate semiquantitative agreement with the numerically exact HEOM results for *k*/*k*_0_ when changing the cavity frequency ω_c_, the light–matter coupling strength η_c_, or the cavity lifetime τ_c_.

Despite the fact that we have demonstrated encouraging progress to obtain the correct VSC resonance condition ω_c_ = ω_0_ with a variety of MQC simulation approaches, the numerical results of *k*/*k*_0_ overestimate the exact results by 3–8 times, depending on the detailed MQC methods. Note that the current quantum and classical subsystem partitions are *not unique*, and one can pick and choose which DOF can be further included inside the quantum subsystem. One can, in principle, use the eq 2 as the Hamiltonian, and include  inside the quantum subsystem by using the Fock state basis. This will, in principle, bring additional accuracy because it also treats the cavity mode quantum mechanically in MQC simulations. In a photoinduced proton-coupled electron transfer (PI–PCET) model,^42^ we have found that this treatment of the proton coordinate indeed brings additional accuracy compared to the classical treatment of the same coordinate. Alternatively, one can quantize  as a ring polymer (imaginary time path integral) and still keep the key vibrational states described at the state level and use nonadiabatic RPMD to simulate the dynamics. In a polariton-mediated electron transfer (PMET) model,^43^ we found that this treatment (quantizing  as a ring polymer and describe the electron donor and acceptor at the state level) gives very accurate rate constant for PMET rate constant. Other more accurate trajectory-based nonadiabatic dynamics methods, such as spin-mapping variable-based partial-linearized density matrix method (spin-PLDM)^44,45^ or mapping-based surface hopping approach^46−48^ should also be able to generate more accurate results. Furthermore, instead of computing population dynamics and then fitting it to extract the rate constant, one can directly obtain the rate constant by computing the flux-side correlation function using trajectory-based methods.^49−51^

The appealing computational cost of all MQC-based methods will enable large-scale simulations that involve many molecules collectively coupled to many cavity modes. The actual experimental condition involves *N* ≈ 10^6^–10^12^ molecules collectively coupled to many cavity modes that obey the dispersion relation  for a Fabry–Pérot cavity, where *k*_∥_ is the continuous in-plane photonic momentum and *k*_⊥_ is the quantized photonic momentum that is perpendicular to the mirror surface. Experimentally, only when ω_**k**_ = ω_0_ at *k*_∥_ = 0 is satisfied (commonly termed the normal incidence) can one observe VSC modification on the rate constant. For a given finite *k*_∥_, it is possible for ω_**k**_ = ω_0_, but there will be no apparent VSC effect.^6,52,53^ Currently, only a few theories can tentatively explain such a resonance condition at the normal incidence.^54,55^ The MQC approaches are thus ideal for simulating this realistic scenario of many molecules coupled to many photon modes inside the Fabry–Pérot cavity and can be used to directly investigate the fundamental mechanism of VSC-modified rate constants. Finally, it would be ideal to simulate the realistic reactive molecule coupled to the solvent DOF inside the cavity and investigate the VSC effect. Currently, there are direct atomistic simulations that treat molecular vibrations and cavity mode  classically.^56−58^ A future direction is to simulate the VSC dynamics with the quantum state description for reactive vibrational DOF  and the rest of DOFs are classical, using the MQC approaches described in the current work.

## Supporting Information Available

Details of the molecular system, including the table of major parameters; details of the HEOM and MQC (γ-SQC, spin-LSC, Ehrenfest, and GFSH) simulations; details of the sampling, quantum dynamics propagation, and fitting procedure to obtain rate constant from the MQC simulations; some additional results from the MQC simulations (PDF)
