# Supplementary material for: Resonance Enhancement of Vibrational Polariton Chemistry Obtained from the Mixed Quantum-Classical Dynamics Simulations
Source: J Phys Chem Lett. 2023 Dec 6;14(49):11208–16. doi: 10.1021/acs.jpclett.3c02985 (PMC10726371; doi:10.1021/acs.jpclett.3c02985)
Supplement: Supplementary file 1 — jz3c02985_si_001.pdf [file jz3c02985_si_001.pdf]

Supporting Information of
Resonance Enhancement of Vibrational Polariton Chemistry Obtained from the Mixed Quantum-Classical Dynamics Simulations

Deping Hu,† Wenxiang Ying,*,‡ and Pengfei Huo*,‡,¶

†*Center for Advanced Materials Research, Beijing Normal University, Zhuhai 519087, China*

‡*Department of Chemistry, University of Rochester, Rochester, New York 14627, USA*

¶*Institute of Optics, Hajim School of Engineering, University of Rochester, Rochester, New York 14627, USA*

E-mail: wying3@ur.rochester.edu; pengfei.huo@rochester.edu

I. Details of the Molecular Model System

To model how VSC influences chemical reactions, we are particularly interested in the one-dimensional double-well (DW) potential[1,2]

$$V(\hat{R}) = -\frac{M\omega_{\mathrm{b}}^2}{2}\hat{R}^2 + \frac{M^2\omega_{\mathrm{b}}^4}{16E_{\mathrm{b}}}\hat{R}^4, \tag{S1}$$

where M is the effective mass of the reaction coordinate, ω_{b} is the barrier frequency, and E_{b} is barrier height of the DW potential. Note that Eq. S1 assumes a symmetric DW potential.

For the system (reaction coordinate), the corresponding eigenvectors $|\nu_i\rangle$ and eigenenergies E_i are obtained by numerically solving

$$\left(\frac{\hat{P}^2}{2M} + V(\hat{R})\right)|\nu_i\rangle = E_i|\nu_i\rangle, \tag{S2}$$

where $V(\hat{R})$ is expressed in Eq. S1. These vibrational eigenstates are obtained by using the

discrete variable representation (DVR) basis.[3] We *diabatize* the two lowest eigenstates as

$$|\nu_{\rm L}\rangle = \frac{1}{\sqrt{2}}\big(|\nu_0\rangle + |\nu_1\rangle\big), \qquad |\nu_{\rm R}\rangle = \frac{1}{\sqrt{2}}\big(|\nu_0\rangle - |\nu_1\rangle\big), \tag{S3}$$

which leads to two energetically degenerate diabatic states, denoted as $|\nu_{\rm L}\rangle$ and $|\nu_{\rm R}\rangle$ for states localized in the left and right wells, respectively, both with the degenerate energy $E_{\rm L} = (E_1 + E_0)/2$ and a small tunneling splitting $V_{\rm LR} = (E_1 - E_0)/2$. Similarly, for $\{|\nu_2\rangle, |\nu_3\rangle\}$, one can diabatize them and obtain the first excited *diabatic vibrational state* in the left well and right well as follows

$$|\nu'_{\rm L}\rangle = \frac{1}{\sqrt{2}}\big(|\nu_2\rangle + |\nu_3\rangle\big), \qquad |\nu'_{\rm R}\rangle = \frac{1}{\sqrt{2}}\big(|\nu_2\rangle - |\nu_3\rangle\big), \tag{S4}$$

with the degenerate diabatic energy $E_{\rm L'} = (E_3 + E_2)/2$ and the tunneling splitting $V'_{\rm LR} = (E_3 - E_2)/2$. Based on the two diabatic states $|\nu_{\rm L}\rangle$ and $|\nu'_{\rm L}\rangle$ in the left well, we define the quantum vibration frequency of the reactant as

$$\hbar\omega_0 \equiv E_{\rm L'} - E_{\rm L}, \tag{S5}$$

which is directly related to the quantum transition of $|\nu_{\rm L}\rangle \to |\nu'_{\rm L}\rangle$. Note that the spectroscopy measurement (IR or transmission spectra) is also directly related to this frequency.

For a practical calculation, truncation has to be made upon the number of matter states, restricting the dynamics in a relatively low energy subspace while ensuring numerical accuracy. As such, the Hamiltonian and the reaction coordinate have their matrix representations in a truncated Hilbert space. Similarly, we also have the vibrational permanent dipole associated with $|\nu_{\rm L}\rangle$ as $\mu_{\rm LL} = \langle\nu_{\rm L}|\mu(\hat{R})|\nu_{\rm L}\rangle$, as well as for vibrationally excited states $|\nu'_{\rm L}\rangle$ as $\mu_{\rm L'L'} = \langle\nu'_{\rm L}|\mu(\hat{R})|\nu'_{\rm L}\rangle$. These permanent dipoles might be important for computing polariton spectra under ultra-strong coupling regimes. To be specific, the matrix representation for the Hamiltonian and the dipole matrices are expressed in the $\{|\nu_{\rm L}\rangle, |\nu_{\rm R}\rangle, |\nu'_{\rm L}\rangle, |\nu'_{\rm R}\rangle\}$ subspace

as follows,

Table S1: Matrix representation of $\hat{H}_\mathrm{M} = \frac{\hat{P}_0^2}{2M} + V(\hat{R}_0)$ in the 4×4 subspace. The unit of energy is in cm^{-1}.

	$\lvert\nu_\mathrm{L}\rangle$	$\lvert\nu_\mathrm{R}\rangle$	$\lvert\nu'_\mathrm{L}\rangle$	$\lvert\nu'_\mathrm{R}\rangle$
$\lvert\nu_\mathrm{L}\rangle$	0	1.025	0	0
$\lvert\nu_\mathrm{R}\rangle$	1.025	0	0	0
$\lvert\nu'_\mathrm{L}\rangle$	0	0	1189.7	47.68
$\lvert\nu'_\mathrm{R}\rangle$	0	0	47.68	1189.7

where the ground state energy is subtracted. On the other hand, the dipole matrix $\hat{R}$ (which is also the system dissipation mode) and its square $\hat{R}^2$ are also represented in the same 4×4 subspace as follows (with a unit of a.u.),

Table S2: Matrix representation of $\hat{R}$ in the 4×4 subspace. The unit of length is in a.u..

	$\lvert\nu_\mathrm{L}\rangle$	$\lvert\nu_\mathrm{R}\rangle$	$\lvert\nu'_\mathrm{L}\rangle$	$\lvert\nu'_\mathrm{R}\rangle$
$\lvert\nu_\mathrm{L}\rangle$	-41.4	-8.06e-8	-9.14	-0.132
$\lvert\nu_\mathrm{R}\rangle$	-8.06e-8	41.4	0.132	9.14
$\lvert\nu'_\mathrm{L}\rangle$	-9.14	0.132	-32.03	- 1.15e-9
$\lvert\nu'_\mathrm{R}\rangle$	-0.132	9.14	- 1.15e-9	32.03

Table S3: Matrix representation of $\hat{R}^2$ in the 4×4 subspace. The unit of length is in a.u..

	$\lvert\nu_\mathrm{L}\rangle$	$\lvert\nu_\mathrm{R}\rangle$	$\lvert\nu'_\mathrm{L}\rangle$	$\lvert\nu'_\mathrm{R}\rangle$
$\lvert\nu_\mathrm{L}\rangle$	1798.8	-2.414	671.3	1.239
$\lvert\nu_\mathrm{R}\rangle$	-2.414	1798.8	1.239	671.3
$\lvert\nu'_\mathrm{L}\rangle$	671.3	1.239	1109.4	2.414
$\lvert\nu'_\mathrm{R}\rangle$	1.239	671.3	2.414	1109.4

From Table S2, one can read the transition dipole matrix element between $\lvert\nu_\mathrm{L}\rangle$ and $\lvert\nu'_\mathrm{L}\rangle$ as $R_{\mathrm{LL}'} = \langle\nu_\mathrm{L}\lvert\hat{R}\rvert\nu'_\mathrm{L}\rangle = -9.14$. Similarly, $R_\mathrm{LL} = -41.4$, and $R_{\mathrm{L'L'}} = -32.03$.

Fig. 1a of the main text provides a schematic illustration of the ground state chemical reaction model (single molecule) and the first few vibrational states of the DW model, denoted as $\lvert\nu_\mathrm{L}\rangle$, $\lvert\nu_\mathrm{R}\rangle$, $\lvert\nu'_\mathrm{L}\rangle$, $\lvert\nu'_\mathrm{R}\rangle$. The pink arrows indicate the potential effect of the cavity modifying vibrational state transitions, and the gray arrow right above the barrier denotes to the fast dissipative tunneling process from $\lvert\nu'_\mathrm{L}\rangle$ to $\lvert\nu'_\mathrm{R}\rangle$. Here, we use the parameters

$E_\mathrm{b} = 2250$ cm^{-1}, and $\hbar\omega_\mathrm{b} = 1000$ cm^{-1}.[4] The eigenstates are obtained with the sinc-DVR basis with 1001 grid points in the range of $-100 \leq R \leq 100$, then diabatized according to Eq. S3 and S4. Note that because $|\nu'_\mathrm{L}\rangle$ and $|\nu'_\mathrm{R}\rangle$ are very close to the top of the barrier, they are not as well localized as $|\nu_\mathrm{L}\rangle$ and $|\nu_\mathrm{R}\rangle$. To be more clear, we briefly summarize the major parameters for the system degrees of freedom (DOF) in Table S4.[4]

Table S4: Table of major parameters

Parameters of system DOF	Notation	Value
Effective mass of the reaction coordinate	M	1 a.u.
Barrier height	E_b	2250 cm^{-1}
Barrier frequency	$\hbar\omega_\mathrm{b}$	1000 cm^{-1}
Vibration frequency (or resonance frequency)	$\hbar\omega_0$	1190 cm^{-1}
Tunneling splitting between $|\nu_\mathrm{L}\rangle$ and $|\nu_\mathrm{R}\rangle$	Δ	1.03 cm^{-1}
Tunneling splitting between $|\nu'_\mathrm{L}\rangle$ and $|\nu'_\mathrm{R}\rangle$	Δ'	47.68 cm^{-1}

II. Exact Quantum Dynamics Simulations of the lossy cavity, and the rate constant

In Fig. 2a of the main text, we use the numerically exact hierarchical equations of motion (HEOM) approach[5–8] to propagate the quantum dynamics of the VSC model. The single molecule, single mode Hamiltonian is expressed as

$$\hat{H} = \frac{\hat{P}_0^2}{2M} + \hat{V}(\hat{R}_0) + \hat{H}_\nu + \hat{H}_\mathrm{eff}, \tag{S6}$$

where $\hat{H}_\nu$ is the dissipative system-bath Hamiltonian that describes the linear coupling between reaction coordinate $\hat{R}$ and phonon bath, expressed as follows

$$\hat{H}_\nu = \frac{1}{2}\sum_i \left[\hat{p}_i^2 + \omega_i^2\left(\hat{x}_i - \frac{c_i}{\omega_i^2}\hat{R}_0\right)^2\right], \tag{S7}$$

and described by the Drude-Lorentz spectral density function,

$$J_\nu(\omega) \equiv \frac{\pi}{2}\sum_j \frac{c_j^2}{\omega_j}\delta(\omega-\omega_j) = \frac{2\lambda_\nu\gamma_\nu\omega}{\omega^2+\gamma_\nu^2}. \tag{S8}$$

We follow Ref. 4 by taking $\gamma_\nu = 200$ cm^{-1} and $\lambda_\nu = 0.1\omega_\mathrm{b}\gamma_\nu/2$. The cavity and its associated loss are combined as

$$\hat{H}_\mathrm{eff} = \frac{1}{2}\sum_j \left[\hat{\tilde{P}}_j^2 + \tilde{\Omega}_j^2\left(\hat{\tilde{X}}_j - \frac{\tilde{C}_j}{\tilde{\Omega}_j^2}\hat{R}_0\right)^2\right], \tag{S9}$$

with the effective spectral density function[9,10] expressed as below

$$J_\mathrm{eff}(\omega) \equiv \frac{\pi}{2}\sum_j \frac{\tilde{C}_j^2}{\tilde{\Omega}_j}\delta(\omega-\tilde{\Omega}_j) = \frac{2\alpha\eta_\mathrm{c}^2\omega_\mathrm{c}^3\omega}{(\omega_\mathrm{c}^2-\omega^2)^2+\alpha^2\omega^2}, \tag{S10}$$

where $\tau_\mathrm{c} = 1/\alpha$ is the cavity lifetime, η_c is the light-matter coupling strength, which varies from 3.125×10^{-4} to 2.5×10^{-3}, a similar range of parameters as used in Ref. 4. In Eq. S10, we explicitly take the Markovian limit for the photon-loss bath, so that the effective spectral density is in the form of a Brownian oscillator. For a practical calculation, truncation has to be made upon the number of matter states, restricting the dynamics in a relatively low-energy subspace while ensuring numerical accuracy. Here, we treat the number of the vibrational eigenstates $\mathcal{F}$ as a convergence parameter of the VSC dynamics. We found that $\mathcal{F} = 4$ will be able to provide accurate k/k_0 compared to the fully converged results when $\mathcal{F} \geq 6$. Fig. S1 presents the HEOM results of k/k_0 as a function of ω_c, with $\mathcal{F} = 2$ to $\mathcal{F} = 10$. One can clearly see that $\mathcal{F} = 4$ provides the converged trend, and $\mathcal{F} = 6$ is fully converged.

For the HEOM propagation, there are several convergence control parameters, including (1) the number of bath terms obtained from the decomposition of the bare environment time-correlation function (TCF), (2) the time step for integration, (3) the depth of the EOMs (or the number of tiers), and (4) on-the-fly filtering[11] error tolerance. We have carefully checked all of the above convergence parameters. More specifically, we use the fourth order

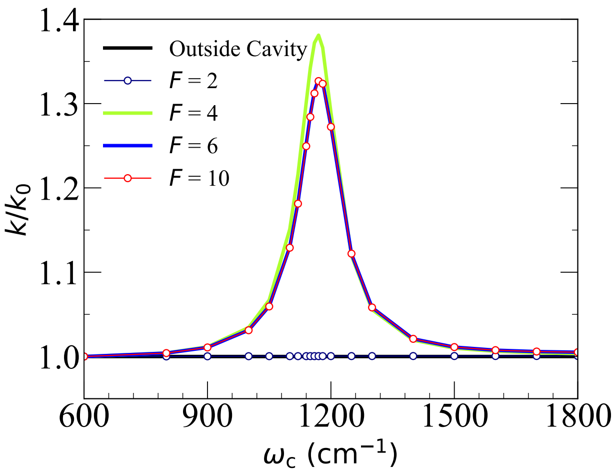

Figure S1: Population dynamics of the four key vibrational states, with (a) $|\nu_\mathrm{L}\rangle$ (red) and $|\nu_\mathrm{L}\rangle$ (green), and (b) $|\nu'_\mathrm{L}\rangle$ (blue) and $|\nu'_\mathrm{R}\rangle$ (magenta). Thick solid lines represent the dynamics that occur outside the cavity, and thin line with open circles represent the dynamics that occur inside the cavity with a coupling strength $\eta_\mathrm{c} = 1.25 \times 10^{-3}$ a.u.

Runge-Kutta (RK-4) integrator with a time step of 0.025 fs, together with the on-the-fly filtering algorithm[11] with an error tolerance of 1×10^{-7}.

The HEOM method requires a factorizable initial condition between the system and bath subspaces. Note that the choice of a particular initial condition will not influence the rate dynamics or rate constant.[2,12] We thus assume a factorizable initial full-density matrix as

$$\hat{\rho}(0) = |\nu_\mathrm{L}\rangle\langle\nu_\mathrm{L}| \otimes \frac{e^{-\beta \hat{h}_\mathrm{B}^\mathrm{eff}}}{Z_\mathrm{B}}, \tag{S11}$$

where $Z_\mathrm{B} \equiv \mathrm{Tr}_\mathrm{B}[e^{-\beta \hat{h}_\mathrm{B}^\mathrm{eff}}]$ is the bath partition function, $\mathrm{Tr}_\mathrm{B}[\cdot]$ denotes taking the partial trace over the bath DOFs, and

$$\hat{h}_\mathrm{B}^\mathrm{eff} = \frac{1}{2}\sum_i \left(\hat{p}_i^2 + \omega_i^2 \hat{x}_i^2\right) + \frac{1}{2}\sum_j \left(\hat{\tilde{P}}_j^2 + \tilde{\Omega}_j^2 \hat{\tilde{X}}_j^2\right) \tag{S12}$$

is the bare effective bath Hamiltonian. We adopt the initial condition of Eq. S11 in all of our numerical simulations with HEOM. The reduced density matrix of the system, on the other hand, is defined as

$$\hat{\rho}_\mathrm{S}(t) = \mathrm{Tr}_\mathrm{B}\left[\hat{\rho}(t)\right], \tag{S13}$$

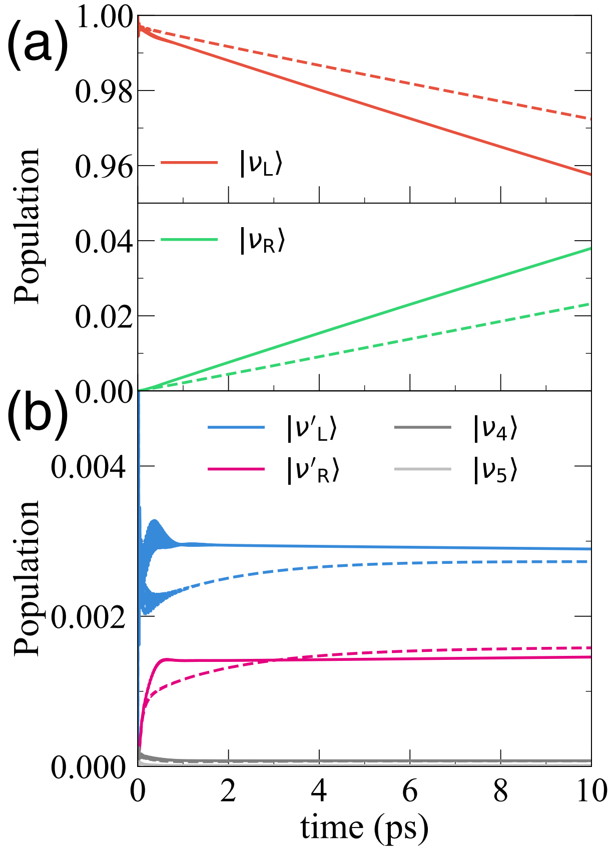

Figure S2: Population dynamics of the first few vibrational states calculated by HEOM. Here we fix $\eta_c = 1.25 \times 10^{-3}$ a.u. and $\tau_c = 200$ fs. Dashed lines denote the results outside the cavity, solid lines denote the results inside the resonant cavity.

where $\hat{\rho}(t)$ is the full density matrix at time t.

Fig. S2 presents the population dynamics of the vibrational states outside the cavity (dashed lines) and coupled to a resonant cavity for $\omega_c = \omega_0 = 1190\ \text{cm}^{-1}$ (solid lines). The temperature is $T = 300$ K, the light-matter coupling strength is $\eta_c = 0.00125$ a.u., and the cavity lifetime is set to be $\tau_c = 200$ fs (in line with the cavity used in the typical VCS experiments[13]). The initial condition is described in Eq. S11, which corresponds to a thermally activated process of the system by the environment. The populations of the six lowest vibrational states are presented, including $|\nu_L\rangle$ (red) and $|\nu_R\rangle$ (green) in panel a, $|\nu'_L\rangle$ (blue) and $|\nu'_L\rangle$ (magenta), $|\nu_4\rangle$ (dark gray), $|\nu_5\rangle$ (light gray) in panel b. Fig. S2a presents the population dynamics of $|\nu_L\rangle$ state (the initially populated state). One can clearly see that when the resonant cavity mode ($\omega_c = \omega_0 = 1190\ \text{cm}^{-1}$) is coupled to the molecular

vibrations (solid line), the population of $|\nu_\mathrm{L}\rangle$ decays much faster than the cavity-free case (dashed line). Fig. S2b shows the population dynamics of the other four vibrational states.

In order to evaluate the forward rate constant, we follow the previous work[2,4,14] by defining the time-dependent reactant ($\mathcal{R}$) and product state ($\mathcal{P}$) populations as

$$P_\mathcal{R}(t) = \mathrm{Tr_S}\left[(1-\hat{h})\hat{\rho}_\mathrm{S}(t)\right], \tag{S14a}$$

$$P_\mathcal{P}(t) = 1 - P_\mathcal{R}(t), \tag{S14b}$$

where the trace $\mathrm{Tr_S}$ in Eq. S14a is performed along the system DOF (which is the reaction coordinate R for the model considered here). In Eq. S14, $\hat{h} = h(\hat{R} - R^\ddagger)$ is the Heaviside operator that projects onto the product states, where $h(R) = 1$ for $R > R^\ddagger$ (in the product region) and $h(R) = 0$ for $R < R^\ddagger$ (in the reaction region), $R^\ddagger$ is the dividing surface. For the symmetric DW model considered here, we use $R^\ddagger = 0$. Under the system's eigen-representation $\{|\nu_i\rangle\}$ in the truncated $\mathcal{F}$-dimensional Hilbert subspace, it is evaluated as

$$P_\mathcal{R}(t) = \sum_{j=1}^{\mathcal{F}} \langle\nu_j|(1-\hat{h})\hat{\rho}_\mathrm{S}(t)|\nu_j\rangle = \sum_{i,j=1}^{\mathcal{F}} \langle\nu_j|(1-\hat{h})|\nu_i\rangle \cdot [\hat{\rho}_\mathrm{S}]_{ij}(t),$$

where $[\hat{\rho}_\mathrm{S}]_{ij}(t) = \langle\nu_i|\hat{\rho}_\mathrm{S}(t)|\nu_j\rangle$ is the system reduced density matrix element, and $\langle\nu_j|(1-\hat{h})|\nu_i\rangle$ is evaluated as

$$\begin{aligned}\langle\nu_j|(1-\hat{h})|\nu_i\rangle &= \int_{-\infty}^{+\infty} dR\ \psi_j^*(R)[1-h(R-R^\ddagger)]\psi_i(R) \\ &= \int_{-\infty}^{R^\ddagger} dR\ \psi_j^*(R)\psi_i(R),\end{aligned}$$

where $\psi_i(R) = \langle R|\nu_i\rangle$, $\psi_j^*(R) = \langle\nu_j|R\rangle$ are the eigenfunctions of the vibrational eigenstates in the position representation (obtained using DVR grid-based method). The forward rate

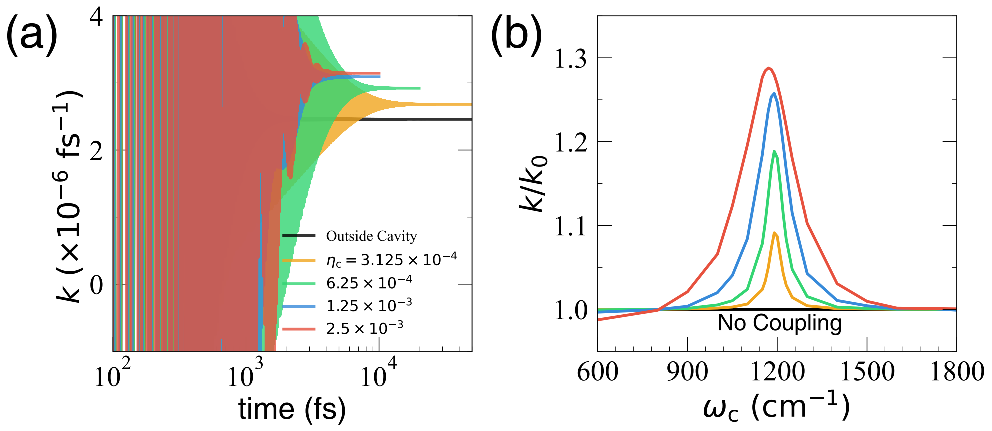

Figure S3: (a) The flux-side correlation functions under various light-matter coupling strengths η_c inside a resonant cavity, calculated using the exact HEOM dynamics. The cavity lifetime is fixed at $\tau_c = 2000$ fs. (b) Same as Fig. 3a of the main text.

constant is then evaluated via[2,10,14,15]

$$k = -\lim_{t \to t_p} \frac{\dot{P}_{\mathcal{R}}(t)}{P_{\mathcal{R}}(t) + \chi_{eq} \cdot [P_{\mathcal{R}}(t) - 1]}, \tag{S15}$$

where $\chi_{eq} \equiv P_{\mathcal{R}}/P_{\mathcal{P}}$ denotes the ratio of equilibrium population between the reactant and product. The time derivative $\dot{P}_{\mathcal{R}}(t)$ is evaluated numerically.

Fig. S3 presents the flux-side correlation functions and the VSC-modified rate profile as a function of the cavity frequency ω_c. Here, the light-matter coupling strength η_c and the cavity frequency ω_c are variables. The temperature is set to be $T = 300$ K, and the cavity lifetime is chosen to be $\tau_c = 2000$ fs. Fig. S3a presents the time-dependent rate constant $k(t)$ (Eq. S15) inside a resonant cavity (with $\omega_c = \omega_0 = 1190$ cm^{-1}). As one gradually increases the light-matter coupling strength η_c (see legend in panel a) from 0 (black, outside the cavity) to 0.0003125 (yellow), 0.000625 (green), 0.00125 (blue), and 0.0025 (red), there is always a well-defined plateau value that provides a rate constant (associated with a rate process), with the plateau time t_p ranging from 4 to 20 ps depending on the specific η_c values. Smaller η_c will lead to longer plateau time t_p. Fig. S3b presents the resonance effect of the VSC-modified rate constant when a molecular vibration is coupled to a cavity. A resonantly enhanced sharp peak of the rate constant is exhibited when $\omega_c = \omega_0$. In panel (b), we present

the rate constant enhancement factor k/k_0, where k and k_0 are obtained from the plateau value of the $k(t)$ in Fig. S3a after reaching the dynamic plateau time.

III. Mixed Quantum-Classical (MQC) Methods

The model Hamiltonian we investigated in this work is in the diabatic representation. As such, we can directly use a lot of trajectory-based non-adiabatic dynamics approaches in their diabatic representation. In the strict diabatic basis $\{|a\rangle\}$ (in the sense that $\langle a|\nabla|b\rangle = 0$ for all $|a\rangle$ and $|b\rangle$), the total Hamiltonian can be expressed as

$$\hat{H} = \frac{1}{2}\hat{\mathbf{P}}^2 + \sum_a U_{aa}(\hat{\mathbf{R}})|a\rangle\langle a| + \sum_{a\neq b} U_{ab}(\hat{\mathbf{R}})|a\rangle\langle b| + U_0(\mathbf{R}), \tag{S16}$$

where $U_{ab}(\hat{\mathbf{R}}) = \langle a|\hat{U}(\hat{\mathbf{R}})|b\rangle$ are the matrix elements of the state-dependent part of the Hamiltonian, $U_0(\mathbf{R})$ is the state independent part of the potential, and $\hat{\mathbf{R}}$ referees to the classical DOF in the classical subsystem. We define the state-dependent potential operator as follows

$$\hat{U}(\hat{\mathbf{R}}) \equiv \sum_a U_{aa}(\hat{\mathbf{R}})|a\rangle\langle a| + \sum_{a\neq b} U_{ab}(\hat{\mathbf{R}})|a\rangle\langle b| \tag{S17}$$

Note that this is not to be confused with $\hat{R}_0$, the reaction coordinate in $\hat{V}$ which we treated quantum mechanically. In this work, we treat the vibrational diabatic states as the quantum subsystem,

$$|a\rangle \in \{|\nu_\mathrm{L}\rangle, |\nu'_\mathrm{L}\rangle, |\nu'_\mathrm{R}\rangle, |\nu_\mathrm{R}\rangle\}, \tag{S18}$$

and we treat the nuclear phonon modes $\{\hat{x}_i\}$ in $\hat{H}_\nu$ (Eq. 3 of the main text) as well as the photonic normal modes $\{\hat{\tilde{X}}_j\}$ in $\hat{H}_\mathrm{eff}$ (Eq. 5 of the main text) as the classical subsystem, such that

$$\hat{\mathbf{R}} \in \{\hat{x}_i, \hat{\tilde{X}}_j\}. \tag{S19}$$

In particular, for the model Hamiltonian in Eq. S6, we have

$$U_{ab}(\hat{\mathbf{R}}) = \langle a|\hat{V}(\hat{R})|b\rangle - \Big(\sum_i c_i\hat{x}_i + \sum_j \tilde{C}_j\hat{\tilde{X}}_j\Big)\cdot\langle a|\hat{R}|b\rangle + \frac{1}{2}\Big(\sum_i \frac{c_i^2}{\omega_i^2} + \sum_j \frac{\tilde{C}_j^2}{\tilde{\Omega}_i^2}\Big)\cdot\langle a|\hat{R}^2|b\rangle \quad \text{(S20)}$$

for the state-dependent potential, where $\hat{R}$ is the reaction coordinate that described quantum mechanically using basis in Eq. S18, and the state-independent parts of the Hamiltonian are

$$U_0(\hat{\mathbf{R}}) = \frac{1}{2}\sum_i \omega_i^2\hat{x}_i^2 + \frac{1}{2}\sum_j \tilde{\Omega}_j^2\hat{\tilde{X}}_j^2 \quad \text{(S21)}$$

$$\frac{1}{2}\hat{\mathbf{P}}^2 = \frac{1}{2}\sum_i \hat{p}_i^2 + \frac{1}{2}\sum_j \hat{\tilde{P}}_j^2 \quad \text{(S22)}$$

Below, we briefly outline the γ-SQC and spin-LSC approach, Ehrenfest dynamics, as well as the global-flux algorithm for trajectory surface hopping (GFSH).

Mapping Formalism. The Meyer-Miller-Stock-Thoss (MMST) formalism[16–18] maps the discrete quantum DOF (described as discrete states) onto continuous phase space variables. In the non-adiabatic mapping approach, the Hamiltonian operator in Eq. S16 is transformed into the following MMST Hamiltonian

$$\mathcal{H}_{\mathrm{m}} = \frac{1}{2}\mathbf{P}^2 + \frac{1}{2}\sum_{ab} V_{ab}(\mathbf{R})\,(p_a p_b + q_a q_b - 2\gamma_b\delta_{ab}) + U_0(\mathbf{R}) \quad \text{(S23)}$$

where $2\gamma_b$ is viewed as a parameter[19] which specifies the ZPE of the mapping oscillators.[19–22] In principle, $2\gamma_b$ is state-specific and trajectory-specific.[23] The MMST mapping Hamiltonian has been historically justified by Stock and Thoss using harmonic oscillator's raising and lowering operators as the mapping operator.[17,18] Recently, it has been derived using the $SU(N)$ Lie group theory or so-called generalized spin mapping approach.[22]

Classical trajectories are generated based on Hamilton's equations of motion (EOM)

$$\dot{q}_b = \partial\mathcal{H}_\mathrm{m}/\partial p_b; \quad \dot{p}_a = -\partial\mathcal{H}_\mathrm{m}/\partial q_a \tag{S24a}$$

$$\dot{\mathbf{R}} = \partial\mathcal{H}_\mathrm{m}/\partial\mathbf{P}; \quad \dot{\mathbf{P}} = -\partial\mathcal{H}_\mathrm{m}/\partial\mathbf{R} = \mathbf{F}, \tag{S24b}$$

with the nuclear force expressed as

$$\mathbf{F} = -\frac{1}{2}\sum_{ab}\nabla U_{ab}(\mathbf{R})\big(p_a p_b + q_a q_b - 2\gamma_b\delta_{ab}\big) - \nabla U_0(\mathbf{R}). \tag{S25}$$

The above classical EOM for both mapping variables (for the quantum subsystem) and the classical DOFs are propagated using the velocity Verlet algorithm.

The γ-SQC approach. The γ-SQC approach samples the initial electronic condition and estimates the population based on the action-angle variables, $\{\varepsilon_b, \theta_b\}$, expressed as follows

$$\varepsilon_b = \frac{1}{2}\left(p_b^2 + q_b^2\right); \quad \theta_b = -\tan^{-1}\left(\frac{p_b}{q_b}\right). \tag{S26}$$

They are inverse related to the mapping variables as follows

$$q_b = \sqrt{2\varepsilon_b}\cos(\theta_b); \quad p_b = -\sqrt{2\varepsilon_b}\sin(\theta_b), \tag{S27}$$

where ε_b is a positive-definite action variable that is directly proportional to the mapping variables' radius in action space.[23]

The SQC approach calculates the population of electronic state $|b\rangle$, which is to be evaluated as[19]

$$\rho_{bb}(t) = \mathrm{Tr}_\mathbf{R}\left[\hat{\rho}(0)e^{i\hat{H}t/\hbar}|b\rangle\langle b|e^{-i\hat{H}t/\hbar}\right] \approx \int d\boldsymbol{\tau}\rho_\mathrm{W}(\mathbf{P},\mathbf{R})W_a(\boldsymbol{\varepsilon}(0))W_b(\boldsymbol{\varepsilon}(t)),$$

where $\hat{\rho}(0) = \hat{\rho}_\mathbf{R}\otimes|a\rangle\langle a|$ is the initial density operator, $\rho_\mathrm{W}(\mathbf{P},\mathbf{R})$ is the Wigner transform of $\hat{\rho}_\mathbf{R}$ operator for the nuclear DOFs, $\boldsymbol{\varepsilon} = \{\varepsilon_1, \varepsilon_2, ..., \varepsilon_\mathcal{N}\}$ is the positive-definite action

variable vector for $\mathcal{N}$ electronic states,[23] $W_a(\boldsymbol{\varepsilon}) = \delta(\varepsilon_a - (1+\gamma_a)) \prod_{a \neq b} \delta(\varepsilon_b - \gamma_b)$ is the Wigner transformed action variables,[24] and $d\boldsymbol{\tau} \equiv d\mathbf{P} \cdot d\mathbf{R} \cdot d\boldsymbol{\varepsilon} \cdot d\boldsymbol{\theta}$. For practical reasons, the above delta functions in $W_a(\boldsymbol{\varepsilon})$ are broadened using a distribution function (so-called window function) that can be used to bin the resulting electronic action variables in action-space.[19] Here, we used the triangle window[23,24] which is expressed as

$$W_b(\boldsymbol{\varepsilon}) = w_1(\varepsilon_b) \prod_{b' \neq b}^{\mathcal{N}} w_0(\varepsilon_b, \varepsilon_{b'}), \tag{S28}$$

where the window functions are defined as

$$w_1(\varepsilon) = \begin{cases} (2-\varepsilon)^{2-\mathcal{N}}, & 1 < \varepsilon < 2 \\ 0, & \text{else} \end{cases} \tag{S29}$$

and

$$w_0(\varepsilon, \varepsilon') = \begin{cases} 1, & \varepsilon' < 2 - \varepsilon \\ 0, & \text{else,} \end{cases} \tag{S30}$$

and trajectories are assigned to state b at time t if $\varepsilon_b \geq 1$ and $\varepsilon_{b'} < 1$ for all $b' \neq b$.

The time-dependent population of the state $|b\rangle$ is computed with Eq. S28. Using the window function estimator, the total population is no longer properly normalized due to the fraction of trajectories that are outside of any window region at any given time.[25] Thus, the total population must be normalized[25] with the following procedure

$$\rho_{bb}(t) / \sum_{a=1}^{N} \rho_{aa}(t) \to \rho_{bb}(t). \tag{S31}$$

In the γ-SQC approach,[23] it was proposed that the mapping ZPE should be chosen in such a way as to constrain the initial force to be composed purely of the initially occupied state.[23] The basic logic of γ-SQC is to choose an γ_b for each state $|b\rangle$ in every given individual trajectory, such that the initial population is forced to respect the initial electronic excitation

focused onto a single excited state. If the initial electronic state is $|a\rangle$, then

$$\gamma_b = \varepsilon_b - \delta_{ba}, \tag{S32}$$

or equivalently,

$$\delta_{ba} = \varepsilon_b - \gamma_b, \tag{S33}$$

where the $\{\varepsilon_b\}$ are uniformly sampled inside the window function (Eq. S28), and then the γ_b are chosen to satisfy Eq. S33.

These γ_b will be explicitly used in the EOMs in Eqs. S24-S25, and in particular, the nuclear forces are now

$$\mathbf{F} = -\frac{1}{2}\sum_{ab} \nabla V_{ab}(\mathbf{R})\big(p_a p_b + q_a q_b - 2\gamma_b \delta_{ba}\big), \tag{S34}$$

ensuring the initial forces (at $t = 0$) are simply $\mathbf{F} = -\nabla V_{aa}(\mathbf{R})$. Previously, without any adjustments to γ_b, the chosen values for γ_b were only dependent on the windowing function itself, *i.e.*, $\gamma_b = 0.366$ for the square Windows and $\gamma_b = 1/3$ for the triangle windows. With the above γ-correction method,[23] each individual trajectory will have its own state-specific γ_b for state $|b\rangle$ that is completely independent of the choice of window function. This method has been proven to provide very accurate non-adiabatic dynamics in model photo-dissociation problems (coupled Morse potential), as well as outperform FSSH (with decoherence correction) in *ab initio* on-the-fly simulations.[26,27]

The Spin-LSC method. For the spin-LSC approach,[21,22] one chooses a universal ZPE parameter $2\gamma_b = \Gamma$ for all states and trajectories. The spin-LSC population dynamics is calculated as

$$\rho_{bb}(t) = \mathrm{Tr}_{\mathbf{R}}\left[\hat{\rho}_R \otimes |a\rangle\langle a| e^{i\hat{H}t/\hbar}|b\rangle\langle b| e^{-i\hat{H}t/\hbar}\right] \approx \int d\boldsymbol{\tau}\rho_{\mathrm{W}}(\mathbf{P},\mathbf{R})\big[|a\rangle\langle a|\big]_{\mathrm{s}}(0)\cdot\big[|b\rangle\langle b|\big]_{\bar{\mathrm{s}}}(t), \tag{S35}$$

where the population estimators are obtained from the Stratonovich-Weyl transformed electronic projection operators, with the expressions as follows[22]

$$\big[|a\rangle\langle a|\big]_{\mathrm{s}} = \frac{1}{2}(q_a^2 + p_a^2 - \Gamma) \tag{S36a}$$

$$\big[|b\rangle\langle b|\big]_{\bar{\mathrm{s}}} = \frac{\mathcal{N}+1}{2(1+\frac{\mathcal{N}\Gamma}{2})^2}\cdot(q_b^2 + p_b^2) - \frac{1-\frac{\Gamma}{2}}{1+\frac{\mathcal{N}\Gamma}{2}}. \tag{S36b}$$

The parameter Γ is related to the radius of the generalized Bloch sphere r_{s} through $\Gamma = \frac{2}{\mathcal{N}}(r_{\mathrm{s}} - 1)$, where s and $\bar{\mathrm{s}}$ are complementary indices in the Stratonovich-Weyl transform. Among the vast parameter space, one of the best-performing choices[21,22] is when $r_{\mathrm{s}} = r_{\bar{\mathrm{s}}} = \sqrt{\mathcal{N}+1}$, which is referred to as s = W, leading to a ZPE parameter

$$\Gamma = \frac{2}{\mathcal{N}}(\sqrt{\mathcal{N}+1} - 1), \tag{S37}$$

as well as the identical expression of $[|a\rangle\langle a|]_{\mathrm{s}}$ and $[|b\rangle\langle b|]_{\bar{\mathrm{s}}}$ in Eq. S36. We further use the focused initial condition[21,22] that replaces the sampling of the mapping variables in the $d\boldsymbol{\tau}$ integral of Eq. S35 with specific values of the mapping variables, such that $\frac{1}{2}(q_a^2+p_a^2-\Gamma) = 1$ for initially occupied state $|a\rangle$ and $\frac{1}{2}(q_b^2 + p_b^2 - \Gamma) = 0$ for the initially unoccupied states $|b\rangle$. The angle variables $\{\theta_b\}$ (Eq. S26) are randomly sampled[22] in the range of $[0, 2\pi)$.

Ehrenfest Dynamics. In the Ehrenfest dynamics, the wavefunction of the quantum subsystem (electronic-photonic DOFs) is written as

$$|\Psi(t)\rangle = \sum_a c_a(t)|a\rangle, \tag{S38}$$

where $|a\rangle$ is the diabatic basis of the quantum subsystem (see Eq. S17). The quantum subsystem is described by the time-dependent Schödinger equation (TDSE):

$$i\hbar\frac{\partial}{\partial t}|\Psi(t)\rangle = \hat{U}(\mathbf{R}(t))|\Psi(t)\rangle, \tag{S39}$$

and the corresponding coefficient equation is

$$i\hbar\frac{\partial}{\partial t}c_a = \sum_b U_{ab}(\mathbf{R}(t))\cdot c_b, \tag{S40}$$

The classical subsystem (nuclear DOF) is propagated using Newton's EOM, where the nuclear force is evaluated from the time-dependent average potential (mean-field)

$$\mathbf{F}(t) = -\langle\Psi(t)|\hat{U}|\Psi(t)\rangle - \nabla U_0(\mathbf{R}(t)) = -\sum_{ab} c_a^*(t)c_b(t)\nabla U_{ab}(\mathbf{R}(t)) - \nabla U_0(\mathbf{R}(t)). \tag{S41}$$

We use the fourth-order Runge-Kutta method to integrate the propagation of the quantum subsystem in Eq. S40. The classical subsystem (nuclear DOF) is propagated using Newton's EOM with the velocity Verlet algorithm.

Trajectory Surface Hopping Approach. In the trajectory surface hopping (TSH) dynamics, we solve the same TDSE for the quantum subsystem as described in Eq. S40. In the TSH dynamics,[28] the nuclear force comes from *only one* specific adiabatic state $|\mathcal{E}_I(\mathbf{R}(t))\rangle$, commonly referred to as the active state. These adiabatic states are the eigenstates of $\hat{U}(\mathbf{R})$ (see Eq. S17) defined as follows

$$\hat{U}(\mathbf{R})|\mathcal{E}_I(\mathbf{R})\rangle = \mathcal{E}_I(\mathbf{R})|\mathcal{E}_I(\mathbf{R})\rangle, \tag{S42}$$

where $|\mathcal{E}_I(\mathbf{R})\rangle = \sum_a |a\rangle\langle a|\mathcal{E}_I(\mathbf{R})\rangle$. At each configuration $\mathbf{R}(t)$, the adiabiatic energy $\mathcal{E}_I(\mathbf{R})$ and expansion coefficients $\langle a|\mathcal{E}_I(\mathbf{R})\rangle$ are obtained by diagonalizing the matrix of $\hat{U}(\mathbf{R})$ with matrix elements $\hat{U}_{ab}(\mathbf{R})$. The nuclear force on the active state $|\mathcal{E}_I(\mathbf{R})\rangle$ is thus

$$\mathbf{F} = -\nabla\mathcal{E}_I(\mathbf{R}), \tag{S43}$$

where $\mathcal{E}_I(\mathbf{R})$ is the energy of the *active* adiabatic polariton state, and I is the active state index determined with the TSH algorithm, which will be determined at every nuclear prop-

agation step. The nuclear gradient is calculated as

$$\nabla \mathcal{E}_I(\mathbf{R}) = \langle \mathcal{E}_I(\mathbf{R})|\nabla \hat{U}|\mathcal{E}_I(\mathbf{R})\rangle, \tag{S44}$$

as the results of the Hellman-Feynman theorem, which can be further expressed as

$$\nabla \mathcal{E}_I = \sum_{ab} \langle \mathcal{E}_I(\mathbf{R})|a\rangle\langle a|\nabla \hat{U}|b\rangle\langle b|\mathcal{E}_I(\mathbf{R})\rangle = \sum_{ab} \langle \mathcal{E}_I(\mathbf{R})|a\rangle \nabla U_{ab}(\mathbf{R})\langle b|\mathcal{E}_I(\mathbf{R})\rangle. \tag{S45}$$

To further obtain the switching probability of the molecular system from one polariton state to another polariton state, we follow the recently developed global flux surface hopping (GFSH) algorithm.[29] This algorithm is shown to outperform the original fewest switches algorithm[28] for systems with more than two electronic states, and for the super-exchange process which is essentially the vibrational dynamics we investigated here (see Fig. S2, where $|\nu'_{\mathrm{L}}\rangle$ and $|\nu'_{\mathrm{R}}\rangle$ acting as super exchange virtual states). Here, we briefly describe how to apply it. First, we express the density matrix element in the *polariton basis* as follows

$$\rho_{IJ}(t) = \langle \mathcal{E}_I(\mathbf{R})|\Psi(t)\rangle\langle \Psi(t)|\mathcal{E}_J(\mathbf{R})\rangle = \sum_{ab} c_a(t)c_b^*(t)\langle \mathcal{E}_I(\mathbf{R})|a\rangle\langle b|\mathcal{E}_J(\mathbf{R})\rangle, \tag{S46}$$

We employ the GFSH algorithm[29] to calculate the probability of switching from the active polariton state $|\mathcal{E}_I\rangle$ to *any other* polariton state $|\mathcal{E}_J\rangle$ during the time interval between t and $t+\delta t$ as follows

$$f_{IJ} = \frac{\Delta\rho_{JJ}}{\rho_{II}} \frac{\Delta\rho_{II}^{\mathrm{pl}}}{\sum_{K\in A}\Delta\rho_{KK}} \quad (\text{if } I\in A \text{ and } J\in B), \tag{S47}$$

where $\Delta\rho_{II} = \rho_{II}(t+\delta t) - \rho_{II}(t)$. From time t to $t+\delta t$, all the adiabatic states that lose population form group A, while all the adiabatic states that gain population form group B. Here, we only need to calculate the switching probability when the current active state $|\mathcal{E}_I\rangle$ belongs to A, and the destination state belongs to B. All other types of state switches, for example, $|\mathcal{E}_I\rangle$ and $|\mathcal{E}_J\rangle$ belong to the same subgroup, or $|\mathcal{E}_I\rangle$ belongs to B and $|\mathcal{E}_J\rangle$ belongs to A, are not allowed, and the switching probabilities are set to 0 based on the algorithm.[29]

The non-adiabatic transition, *i.e.* stochastic switches from the currently occupied state $|\mathcal{E}_I\rangle$ to another state $|\mathcal{E}_K\rangle$, occurs if the following condition is satisfied

$$\sum_{J=1}^{K} f_{IJ} < \zeta < \sum_{J=1}^{K+1} f_{IJ}, \tag{S48}$$

where ζ is a uniform randomly generated number between 0 and 1 at each nuclear time step. If the transition is accepted, the active state is set to the new adiabatic state $|\mathcal{E}_K\rangle$.

For each nuclear time step, the quantum subsystem is propagated using Eq. S40. We calculate the hopping probabilities and assess if the system should hop to another state for both of these two steps. If a hopping event happens from current state $|\mathcal{E}_I\rangle$ to new $|\mathcal{E}_K\rangle$ due to quantum subsystem evolution itself governed by Eq. S40, the velocities of the nuclei are rescaled along the direction of the NAC $\mathbf{d}_{IK}(\mathbf{R})$ in order to conserve the total energy.[30] In particular, the NAC between two polaritonic states can be expressed as[30]

$$\mathbf{d}_{IJ} = \langle\mathcal{E}_I|\nabla|\mathcal{E}_J\rangle = \frac{\langle\mathcal{E}_I|\nabla V|\mathcal{E}_J\rangle}{\mathcal{E}_J - \mathcal{E}_I}. \tag{S49}$$

One can further express Eq. S49 by inserting the completeness relation as

$$\mathbf{d}_{IJ} = \sum_{ab} \frac{\langle\mathcal{E}_I|a\rangle\langle a|\nabla\hat{U}|b\rangle\langle b|\mathcal{E}_J\rangle}{\mathcal{E}_J - \mathcal{E}_I}, \tag{S50}$$

where $\langle a|\nabla\hat{U}|b\rangle = \nabla\langle a|\hat{U}|b\rangle$. For the VSC model system considered here, $\mathbf{d}_{IJ}$ can be directly computed from the analytic expression of $\nabla\langle a|\hat{U}|b\rangle$ and the diabatic-adiabatic expansion coefficients $\langle\mathcal{E}_I|a\rangle$.

When computing the population dynamics in a representation that is *not* the adiabatic states of $\hat{U}$, there is no unique way to calculate them in the TSH approach.[31] In this work, we follow the estimator proposed by Subotnik *et al*,[31] which shows more accurate results in our previous work for polariton dynamics.[32] Below, we briefly introduce this estimator $\tilde{\rho}_{IJ}(\mathbf{R}_l(t))$ for the l_{th} trajectory, where the adiabatic population is calculated using the active

state index, and the off-diagonal elements are computed using the polaritonic state expansion coefficients $\{c_I(t)\}$, as follows

$$\tilde{\rho}_{IJ}(\mathbf{R}_l(t)) = \begin{cases} \delta_{IK}, & I = J \\ c_I c_J^*, & I \neq J, \end{cases} \tag{S51}$$

where K is the active polaritonic state. This estimator was developed in connection with the mixed quantum-classical Liouville equation,[31] and has shown to provide more accurate diabatic population,[31] as well as adiabatic-Fock states populations for a Shin-Metiu model coupled to the cavity.[32]

To get the diabatic state population of the $|a\rangle$ state ρ_{aa} from the TSH simulation, the most straightforward way is through following unitary transformation

$$\rho_{aa}(\mathbf{R}_l(t)) = \langle a|\mathcal{E}_I(\mathbf{R}_l(t))\rangle \tilde{\rho}_{IJ}(t) \langle \mathcal{E}_J(\mathbf{R}_l(t))|a\rangle \tag{S52}$$

The diabatic state population is then obtained from the trajectory average as follows

$$P_a(t) = \frac{1}{N}\sum_{l=1}^{N} \rho_{aa}(\mathbf{R}_l(t)). \tag{S53}$$

where N is the total number of trajectories.

IV. Fitting Procedure to Obtain Rate Constant from the MQC Simulations

In this work, we obtain the rate constant from MQC simulations by fitting the population dynamics. Because the dynamics of $|\nu'_{\mathrm{L}}\rangle$ and $|\nu'_{\mathrm{R}}\rangle$ are almost virtual, the overall dynamics can be viewed as $|\nu_{\mathrm{L}}\rangle \to |\nu_{\mathrm{R}}\rangle$ (mediated by the $|\nu'_{\mathrm{L}}\rangle$ and $|\nu'_{\mathrm{R}}\rangle$ virtual state), and isomorphic to the donor-acceptor type dynamics. We denote the population of these two states as $P_{\mathrm{L}}(t)$ and $P_{\mathrm{R}}(t)$, which are obtained from the MQC simulations.

The $|\nu_{\mathrm{R}}\rangle$ state population is related to the forward rate constant k_{f} and the backward

rate constant k_{b} as follows

$$\frac{dP_{\mathrm{R}}(t)}{dt} = k_{\mathrm{f}} \cdot P_{\mathrm{L}}(t) - k_{\mathrm{b}} \cdot P_{\mathrm{R}}(t). \tag{S54}$$

Integrating the above equation on both sides, we obtain the following relation

$$\int_{P_{\mathrm{R}}(0)}^{P_{\mathrm{R}}(t^{'})} dP_{\mathrm{R}}(t) = k_{\mathrm{f}} \int_{0}^{t^{'}} P_{\mathrm{L}}(t)dt - k_{\mathrm{b}} \int_{0}^{t^{'}} P_{\mathrm{R}}(t)dt, \tag{S55}$$

where $t^{'}$ is varied within the range $[0, t_{\mathrm{f}}]$ when t_{f} is the characteristic time scale for the reaction. The above equation can be further written as

$$P_{\mathrm{R}}(t^{'}) = k_{\mathrm{f}}\tilde{P}_{\mathrm{L}}(t^{'}) - k_{\mathrm{b}}\tilde{P}_{\mathrm{R}}(t^{'}), \tag{S56}$$

where $\tilde{P}_{\mathrm{L}}(t^{'}) = \int_{0}^{t^{'}} P_{\mathrm{L}}(t)dt$, $\tilde{P}_{\mathrm{R}}(t^{'}) = \int_{0}^{t^{'}} P_{\mathrm{R}}(t)dt$, and $P_{\mathrm{L}}(0) = 1$, $P_{\mathrm{R}}(0) = 0$.

With the converged populations $P_{\mathrm{L}}(t)$ and $P_{\mathrm{R}}(t)$ obtained from the MQC simulations, we then numerically integrate them to compute $P_{\mathrm{L}}(t^{'})$ and $P_{\mathrm{R}}(t^{'})$, and obtain k_{f} and k_{b} through fitting the $P_{\mathrm{R}}(t^{'})$ curve in the time-range of $[0, t_{\mathrm{f}}]$. In this work, we use $t_{\mathrm{f}} = 7$ ps, such that other competing dynamical processes do not complicate this simple fitting scheme. We have carefully checked the robustness of this scheme by varying the value of t_{f} and found that the rate obtained is insensitive to the particular choice of t_{f}, as long as avoiding other competing dynamical processes as mentioned above. The same fitting scheme has been previously employed to obtain the rate constant from time-dependent populations.[33,34] Here, we fit both k_{f} and k_{b} in the above equation, without explicitly requiring the relation of the detailed balance.

V. Computational Details

We discretize the spectral density function of $J_\nu(\omega)$ (Eq. S8) based on an algorithm in Ref. 35.

$$\omega_i = \gamma_\nu \tan\left(\frac{i}{N}\tan^{-1}(\omega_{\mathrm{max}}/\gamma_\nu)\right), \tag{S57a}$$

$$c_i = 2\omega_i\sqrt{\lambda_\nu \frac{\tan^{-1}(\omega_{\mathrm{max}}/\gamma_\nu)}{\pi N}}, \tag{S57b}$$

where $\omega_{\mathrm{max}} \gg \gamma_\nu$ is the maximum frequency while discretizing the bath frequencies. Here, we choose $N = 300$ bath modes, $\omega_{\mathrm{max}} = 10\ \gamma_\nu$. The bath parameters are $\gamma_\nu = 200\ \mathrm{cm}^{-1}$ and $\lambda_\nu = 0.1\omega_{\mathrm{b}}\gamma_\nu/2$, where $\omega_{\mathrm{b}} = 1000\ \mathrm{cm}^{-1}$.

On the other hand, the spectral density function $J_{\mathrm{eff}}(\omega)$ in Eq. S10 is sampled based on a procedure introduced in Ref. 36, in which the bath frequencies $\{\tilde{\Omega}_j\}$ are equally spaced by $\Delta\omega$. The value of $\Delta\omega$ is chosen so that the Poincaré recurrence time $2\pi/\Delta\omega$ is long compared to the simulation time. Also, $\Delta\omega$ should be chosen so that a satisfactory detailed sampling of the peak(s) of $J_{\mathrm{eff}}(\omega)$ is captured. Since the width of the peak of $J_{\mathrm{eff}}(\omega)$ is inversely proportional to the cavity lifetime τ_{c}, the $\Delta\omega$ used during the sampling process is determined by the following expression,

$$\Delta\omega = 30/(\tau_{\mathrm{c}}N), \tag{S58}$$

where $N = 300$ is the number of bath modes to represent $J_{\mathrm{eff}}(\omega)$. The resulting coupling coefficients $\tilde{C}_j$ are expressed as follows,[36]

$$\tilde{C}_j = \sqrt{\frac{2}{\pi}J_{\mathrm{eff}}(\tilde{\Omega}_j)\tilde{\Omega}_j\Delta\omega}. \tag{S59}$$

Fig. S4 presents the sampled effective bath modes for the effective spectral density $J_{\mathrm{eff}}(\omega)$ with different cavity lifetime τ_{c}. We can see that, the peak of the effective spectral density

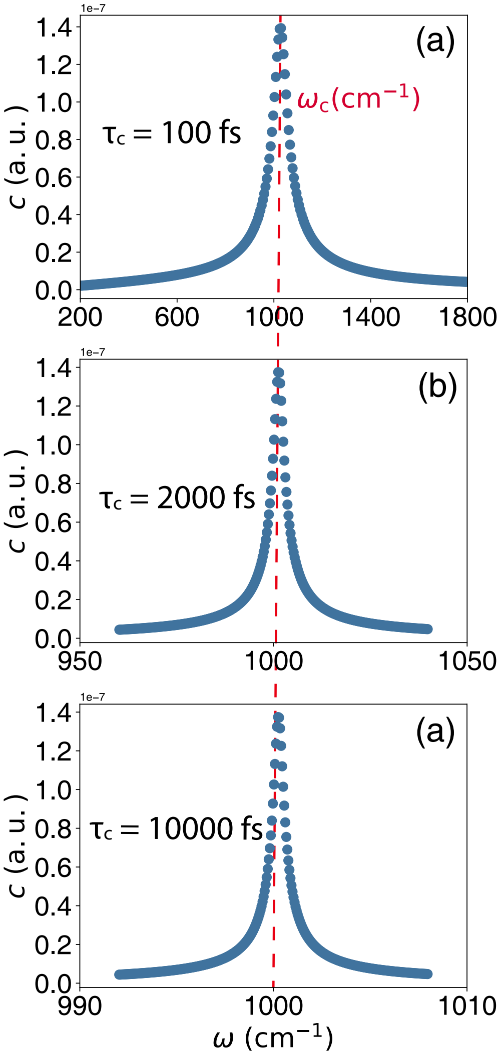

Figure S4: The sampled effective bath modes (frequencies and coefficients) for the effective spectral density $J_{\text{eff}}(\omega)$ with different cavity lifetime τ_c. The coupling strength is $\eta_c = 1.25 \times 10^{-3}$ a.u.. and the cavity frequency is $\omega_c = 1000$ cm^{-1}.

is well sampled for different cavity lifetimes.

The nuclear time step used in all the MQC simulations is $dt_{\mathrm{n}} = 6$ a.u., with 30 electronic time steps for the quantum subsystem integration during each nuclear time step (with $dt_{\mathrm{e}} = 0.2$ a.u.). The population dynamics obtained from all MQC methods were averaged over 20000 trajectories, and the typical trend of convergence will show up with as few as 10^3 trajectories.

The initial conditions for the MQC simulations are chosen to be identical in Eq. S11, which is a tensor product of $|\nu_{\mathrm{L}}\rangle$ and the thermal state for the classical subsystem. The initial conditions for the classical DOFs (including the phonon bath and the effective bath) $\{x_i, p_i, \tilde{X}_j, \tilde{P}_j\}$ are sampled based on the Wigner density $[e^{-\beta \hat{h}_{\mathrm{B}}^{\mathrm{eff}}}]_{\mathrm{W}}$, which is the Wigner transform of the thermal density operator $e^{-\beta \hat{h}_{\mathrm{B}}^{\mathrm{eff}}}$, and $\hat{h}_{\mathrm{B}}^{\mathrm{eff}}$ is a harmonic bath Hamiltonian expressed in Eq. S12.

In the GFSH simulation used in this work, we set $|\nu_{\mathrm{L}}\rangle$ (the vibrational diabatic state in the left well) as the initial state, which corresponds to a thermal initial condition in Eq. S11. Because this is not an adiabatic state, the initial electronic coefficients in the adiabatic representations are obtained through the unitary transformation as $c_I(0) = \langle \mathcal{E}_I(\mathbf{R}(0)) | \nu_{\mathrm{L}} \rangle$, where $\mathbf{R}(0)$ is the sampled initial geometry for the classical subsystems. The initial active state is randomly chosen based on a Monte-Carlo algorithm based on the magnitude of $|c_I(0)|^2$.

VI. Additional Results from the MQC Simulations

Table S5 provides the rate constant of outside cavity k_0 and inside the cavity k (resonant case) with different MQC simulations. For the dynamics inside the cavity, the coupling strength is $\eta_{\mathrm{c}} = 1.25 \times 10^{-3}$ a.u. and the cavity lifetime is $\tau_{\mathrm{c}} = 2000$ fs.

Fig. S5 presents the γ-SQC population dynamics for the outside cavity case, by changing the maximum phonon frequency $\omega_{\max}$ (see Eq. S57a). We found that when $\omega_{\max} < 500$ cm^{-1}, the population dynamics is closer to the HEOM results, but the population dynamics

Table S5: The rate constants outside cavity k_0 and inside the cavity k (resonant case) with different dynamics methods.

Dynamics methods	Outside cavity (10^{-6} fs^{-1})	Inside Cavity (10^{-6} fs^{-1})
HEOM	2.48	3.11
γ-SQC	29.0	99.4
spin-LSC	32.1	91.2
GFSH	64.7	365.3
Ehrenfest	34.1	150.5

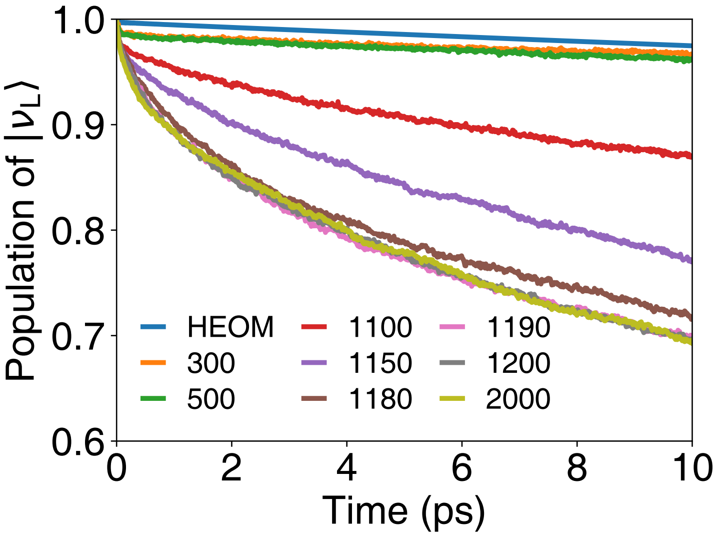

Figure S5: Population dynamics of the $|\nu_{\mathrm{L}}\rangle$ state outside the cavity with different maximum frequencies ($\omega_{\max}$) in the initial sampling for the phonon bath. The HEOM result is also presented for comparison.

does not converge until $\omega_{\max} > 1190$ cm^{-1}. These high phonon frequencies (under room temperature, $k_B T \approx 200$ cm^{-1}) are quantum vibrational modes, and the classical EOM does not properly preserve the zero-point-energy within the Wigner distribution, especially when the model Hamiltonian contains the off-diagonal coupling (see Eq. S20). Our previous work of using non-adiabatic ring polymer MD to simulate polariton-mediated electron transfer[37] suggests that using ring polymer to quantize the cavity mode $\hat{q}_c$ can provide a much more accurate population dynamics for the quantum subsystem.

Fig. S6 presents the population dynamics of the vibrational states from (a-b) Ehrenfest dynamics, (c-d) global flux surface hopping, and (e-f) Spin-LSC approach. The solid lines correspond to the dynamics outside the cavity, and the open circles (with think think-guided line) present the population dynamics inside the cavity (under the resonance condition $\omega_c = \omega_0$). The light-matter coupling strength is $\eta_c = 1.25 \times 10^{-3}$ a.u. and the cavity lifetime is $\tau_c = 2000$ fs. These population dynamics are qualitatively similar to the γ-SQC results presented in Fig. 2 of the main text. However, similar to the γ-SQC results, they overestimate the rate constant by at least 10 times compared to the HEOM results presented in Fig. S2. In particular, the spin-LSC approach provides the most accurate population for the $|\nu_L\rangle$ and $|\nu_R\rangle$ states (see Fig. S6e) among the three methods presented in this figure, and is at the same level of the γ-SQC (see Fig. 2a of the main text). However, spin-LSC does generate negative populations for the $|\nu'_L\rangle$ and $|\nu'_R\rangle$ states (see Fig. S6f) as a feature of the method.

Fig. S7 presents a convergence test of γ-SQC with an increasing number of the vibrational states, with $\mathcal{F} = 4$ states (blue) and $\mathcal{F} = 6$ states (red) for the k/k_0 results as a function of cavity frequency ω_c. The model parameters are exactly the same as in Fig. S1. One can see a similar trend of the convergence as shown in Fig. S1, and the correct resonance enhancement at $\omega_c \approx \omega_0$. The $\mathcal{F} = 4$ states results provide the quantitative agreement with the converged results.

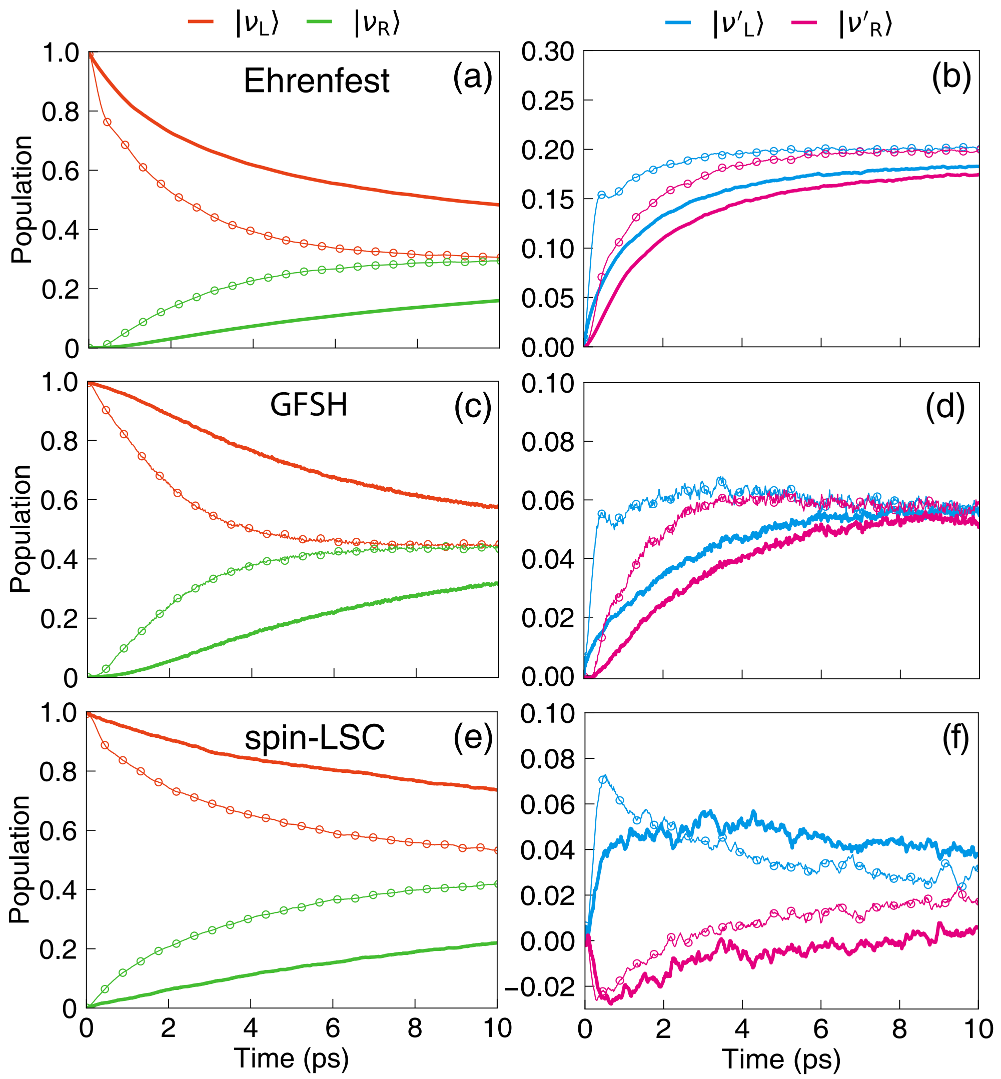

Figure S6: Population dynamics of the four key vibrational states, with (a) $|\nu_{\rm L}\rangle$ (red) and $|\nu_{\rm L}\rangle$ (green), and (b) $|\nu'_{\rm L}\rangle$ (blue) and $|\nu'_{\rm R}\rangle$ (magenta). Thick solid lines represent the dynamics that occur outside the cavity, and thin lines with open circles represent the dynamics that occur inside the cavity with a coupling strength $\eta_{\rm c} = 1.25 \times 10^{-3}$ a.u. and the cavity lifetime $\tau_{\rm c} = 2000$ fs.

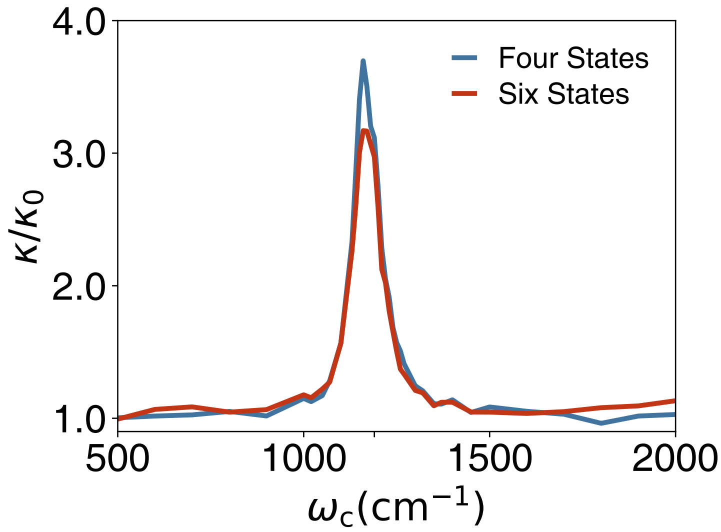

Figure S7: The resonance behavior of k/k_0 as a function of cavity frequency ω_c using γ-SQC simulations with different numbers of key vibrational states. The coupling strength is $\eta_c = 1.25 \times 10^{-3}$ a.u. and the cavity lifetime is $\tau_c = 2000$ fs.

References

(1) Topaler, M.; Makri, N. Quantum rates for a double well coupled to a dissipative bath: Accurate path integral results and comparison with approximate theories. *J. Chem. Phys.* **1994**, *101*, 7500–7519.

(2) Shi, Q.; Zhu, L.; Chen, L. Quantum rate dynamics for proton transfer reaction in a model system: Effect of the rate promoting vibrational mode. *J. Chem. Phys.* **2011**, *135*, 044505.

(3) Colbert, D. T.; Miller, W. H. A novel discrete variable representation for quantum mechanical reactive scattering via the S-matrix Kohn method. *J. Chem. Phys.* **1992**, *96*, 1982–1991.

(4) Lindoy, L. P.; Mandal, A.; Reichman, D. R. Quantum dynamical effects of vibrational strong coupling in chemical reactivity. *Nat. Commun.* **2023**, *14*, 2733.

(5) Tanimura, Y. Nonperturbative expansion method for a quantum system coupled to a harmonic-oscillator bath. *Phys. Rev. A* **1990**, *41*, 6676–6687.

(6) Tanimura, Y. Stochastic Liouville, Langevin, Fokker–Planck, and Master Equation Approaches to Quantum Dissipative Systems. *J. Phys. Soc. Jpn.* **2006**, *75*, 082001.

(7) Xu, R.-X.; Cui, P.; Li, X.-Q.; Mo, Y.; Yan, Y. Exact quantum master equation via the calculus on path integrals. *J. Chem. Phys.* **2005**, *122*, 041103.

(8) Xu, R.-X.; Yan, Y. Dynamics of quantum dissipation systems interacting with bosonic canonical bath: Hierarchical equations of motion approach. *Phys. Rev. E* **2007**, *75*, 031107.

(9) Thoss, M.; Wang, H.; Miller, W. H. Self-consistent hybrid approach for complex systems: Application to the spin-boson model with Debye spectral density. *J. Chem. Phys.* **2001**, *115*, 2991–3005.

(10) Lawrence, J. E.; Fletcher, T.; Lindoy, L. P.; Manolopoulos, D. E. On the calculation of quantum mechanical electron transfer rates. *J. Chem. Phys.* **2019**, *151*, 114119.

(11) Shi, Q.; Chen, L.; Nan, G.; Xu, R.-X.; Yan, Y. Efficient hierarchical Liouville space propagator to quantum dissipative dynamics. *J. Chem. Phys.* **2009**, *130*, 084105.

(12) Bose, A.; Makri, N. Non-equilibrium reactive flux: A unified framework for slow and fast reaction kinetics. *J. Chem. Phys.* **2017**, *147*, 152723.

(13) Thomas, A.; Lethuillier-Karl, L.; Nagarajan, K.; Vergauwe, R. M. A.; George, J.; Chervy, T.; Shalabney, A.; Devaux, E.; Genet, C.; Moran, J.; Ebbesen, T. W. Tilting a ground-state reactivity landscape by vibrational strong coupling. *Science* **2019**, *363*, 615–619.

(14) Wang, H.; Skinner, D. E.; Thoss, M. Calculation of reactive flux correlation functions

for systems in a condensed phase environment: A multilayer multiconfiguration time-dependent Hartree approach. *J. Chem. Phys.* **2006**, *125*, 174502.

(15) Craig, I. R.; Thoss, M.; Wang, H. Proton transfer reactions in model condensed-phase environments: Accurate quantum dynamics using the multilayer multiconfiguration time-dependent Hartree approach. *J. Chem. Phys.* **2007**, *127*, 144503.

(16) Meyer, H.; Miller, W. H. A classical analog for electronic degrees of freedom in nonadiabatic collision processes. *J. Chem. Phys.* **1979**, *70*, 3214–3223.

(17) Stock, G.; Thoss, M. Semiclassical Description of Nonadiabatic Quantum Dynamics. *Phys. Rev. Lett.* **1997**, *78*, 578–581.

(18) Thoss, M.; Stock, G. Mapping Approach to the Semiclassical Description of Nonadiabatic Quantum Dynamics. *Phys. Rev. A* **1999**, *59*, 64–79.

(19) Miller, W. H.; Cotton, S. J. Classical Molecular Dynamics Simulation of Electronically Non-Adiabatic Processes. *Faraday Discuss.* **2016**, *195*, 9–30.

(20) Müller, U.; Stock, G. Flow of zero-point energy and exploration of phase space in classical simulations of quantum relaxation dynamics. II. Application to nonadiabatic processes. *J. Chem. Phys.* **1999**, *111*, 77–88.

(21) Runeson, J. E.; Richardson, J. O. Spin-mapping approach for nonadiabatic molecular dynamics. *J. Chem. Phys.* **2019**, *151*, 044119.

(22) Runeson, J. E.; Richardson, J. O. Generalized spin mapping for quantum-classical dynamics. *J. Chem. Phys.* **2020**, *152*, 084110.

(23) Cotton, S. J.; Miller, W. H. Trajectory-adjusted electronic zero point energy in classical Meyer-Miller vibronic dynamics: Symmetrical quasiclassical application to photodissociation. *J. Chem. Phys.* **2019**, *150*, 194110.

(24) Cotton, S. J.; Miller, W. H. A new symmetrical quasi-classical model for electronically non-adiabatic processes: Application to the case of weak non-adiabatic coupling. *J. Chem. Phys.* **2016**, *145*, 144108.

(25) Cotton, S. J.; Miller, W. H. Symmetrical windowing for quantum states in quasi-classical trajectory simulations: Application to electronically non-adiabatic processes. *J. Chem. Phys.* **2013**, *139*, 234112.

(26) Hu, D.; Xie, Y.; Peng, J.; Lan, Z. On-the-fly symmetrical quasi-classical dynamics with Meyer–Miller mapping Hamiltonian for the treatment of nonadiabatic dynamics at conical intersections. *J. Chem. Theory Comput.* **2021**, *17*, 3267–3279.

(27) Weight, B. M.; Mandal, A.; Huo, P. Ab initio symmetric quasi-classical approach to investigate molecular Tully models. *J. Chem. Phys.* **2021**, *155*, 084106.

(28) Tully, J. C. Molecular dynamics with Electronic Transitions. *J. Chem. Phys.* **1990**, *93*, 1061–1071.

(29) Wang, L.; Trivedi, D.; Prezhdo, O. V. Global Flux Surface Hopping Approach for Mixed Quantum-Classical Dynamics. *J. Chem. Theory Comput.* **2014**, *10*, 3598–3605.

(30) Hammes-Schiffer, S.; Tully, J. C. Proton Transfer in Solution: Molecular Dynamics with Quantum Transitions. *J. Chem. Phys.* **1994**, *12*, 4657–4667.

(31) Landry, B. R.; Falk, M. J.; Subotnik, J. E. Communication: The correct interpretation of surface hopping trajectories: How to calculate electronic Properties. *J. Chem. Phys.* **2013**, *139*, 211101.

(32) Hu, D.; Mandal, A.; Weight, B. M.; Huo, P. Quasi-Diabatic Propagation Scheme for Simulating Polariton Chemistry. *J. Chem. Phys.* **2022**, *157*, 194109.

(33) Jain, A.; Subotnik, J. E. Does Nonadiabatic Transition State Theory Make Sense Without Decoherence? *The Journal of Physical Chemistry Letters* **2015**, *6*, 4809–4814, PMID: 26631360.

(34) Jain, A.; Subotnik, J. E. Surface Hopping, Transition State Theory, and Decoherence. II. Thermal Rate Constants and Detailed Balance. *J. Chem. Phys. 134107 (2015)* **2015**, *143*, 134107.

(35) Huo, P.; Coker, D. F. Semi-classical path integral non-adiabatic dynamics: a partial linearized classical mapping Hamiltonian approach. *Mol. Phys.* **2012**, *110*, 1035–1052.

(36) Hughes, K. H.; Christ, C. D.; Burghardt, I. Effective-mode representation of non-Markovian dynamics: A hierarchical approximation of the spectral density. I. Application to single surface dynamics. *J. Chem. Phys.* **2009**, *131*, 024109.

(37) Chowdhury, S. N.; Mandal, A.; Huo, P. Ring polymer quantization of the photon field in polariton chemistry. *J. Chem. Phys.* **2021**, *154*, 044109.
